# Compressible and Stretchable Aerogels: Construction Strategies and Applications in Personal Thermal Management and Wearable Electronics

**DOI:** 10.3390/gels12090806

**Published:** 2026-09-03

**Authors:** Caixia Ren, Yuping Li, Yongtao Wang, Gangyue Li, Xuepeng Ni, Liyin Hou, Shanshan Guo

**Affiliations:** 1 School of Energy and Constructional Engineering, Shandong Huayu University of Technology, Dezhou 253034, China; rcx@huayu.edu.cn (C.R.); lyp@huayu.edu.cn (Y.L.); ligysuep@126.com (G.L.); 2 Shandong Key Laboratory of Intelligent Manufacturing Technology for Advanced Power Equipment, Weifang University, Weifang 261061, China; yongtao-wang@hotmail.com (Y.W.); guoss@wfu.edu.cn (S.G.)

**Keywords:** flexible aerogels, stretchable aerogels, sensors, personal thermal management, supercapacitors

## Abstract

Mechanically compliant aerogels are increasingly important for wearable systems that require lightweight porous materials to retain function under repeated deformation. However, compressibility and stretchability impose different structural demands and should not be treated as equivalent manifestations of flexibility. This review provides a loading-mode-specific framework of compressible and stretchable aerogels. It summarizes how network chemistry, interfacial interactions, and multiscale architectures govern deformation, recovery, strength, and fatigue resistance under compression and tension. The relationships between these mechanical characteristics and thermal, spectral, and electrical functions are subsequently discussed in the context of wearable personal thermal management sensors, biosensors, and flexible energy-storage devices. Finally, current challenges are summarized in terms of mechanical–functional balancing, long-term durability, and scalable fabrication, providing guidance for the future development of mechanically reliable aerogel-based wearable materials.

## 1. Introduction

The rapid development of wearable electronics, personal thermal management (PTM) systems, and bio-integrated devices has created increasing demand for lightweight and mechanically compliant materials that can maintain their functions under repeated deformation [1,2,3]. Aerogels, featuring highly porous frameworks, low density, low thermal conductivity [4,5], and readily functionalized properties [6], are competitive candidates in these fields. However, conventional aerogels show poor deformability, structural stability, and fatigue resistance in complex stress environments (e.g., compression, bending, and twisting) [4]. These limitations are incompatible with the transition of wearable technologies from rigid platforms toward soft, conformable, and dynamically deformable systems. Therefore, it is essential to create aerogels that can accommodate large deformations while retaining their porous structures and intended functions.

The mechanical behaviors of aerogels are governed jointly by their inherent properties and multiscale architectures. Accordingly, mechanically compliant aerogels can generally be constructed through two complementary routes. The first involves chemical and interfacial engineering, such as introducing organic components [7,8], constructing covalently crosslinked polymer networks [9,10], and strengthening interactions among nanoscale building blocks [11]. The second is architectural regulation, which includes constructing well-ordered porous structures [12], entangled fibrous frameworks [13], and macroscopic arrays through techniques such as freeze casting, three-dimensional (3D) electrospinning, and additive manufacturing. These two strategies provide a common design framework for both compressible and stretchable aerogels, although their specific roles depend strongly on the loading mode. Under compression, deformation can be accommodated through reversible pore closure and skeleton buckling, bending, and rotation, thus enabling aerogels to achieve compressibility, elastic recovery, and cyclic resilience [14]. In contrast, tensile loading directly stresses network junctions and readily induces stress concentration, crack propagation, and structural disconnection [15]. Therefore, stretchability presents a more stringent mechanical challenge, and many highly compressible aerogels exhibit limited stretchability. Achieving high stretchability requires these two routes to specifically target network continuity and tensile stress redistribution. At the chemical and interfacial level, this can be achieved by constructing intrinsically extensible skeletons, robust or dynamic junctions, and energy-dissipating interfaces [15,16]. At the architectural level, geometrically deformable networks can convert macroscopic tension into local bending, rotation, unfolding, or interfacial sliding [17,18], thereby reducing the direct tensile strain imposed on the skeleton.

By integrating loading-mode-specific mechanical design with thermal, electrical, and spectral functionalities, aerogels can support two major categories of wearable applications. In PTM, porous aerogels enable thermal insulation, radiative cooling, heating, and adaptive temperature regulation through heat-transfer control, spectral tuning, and heat storage/release [19,20,21]. In wearable electronics, conductive aerogels prepared from conductive materials allow deformation sensing, biochemical detection, and energy storage under repeated body motion [22,23,24,25]. Many reviews have summarized preparation strategies and applications from different perspectives. For instance, some publications summarize the preparation methods of compressible or stretchable aerogels according to their flexibility mechanisms and further analyze their applications [1,26]. Some reviews emphasize the property optimization of specific aerogels, which are exploited as sensors, flexible storage devices, or electromagnetic interference shielding materials [26,27,28]. In addition, a few reviews focus on the application of flexible aerogels in wearable PTM [5].

**Figure 1:**
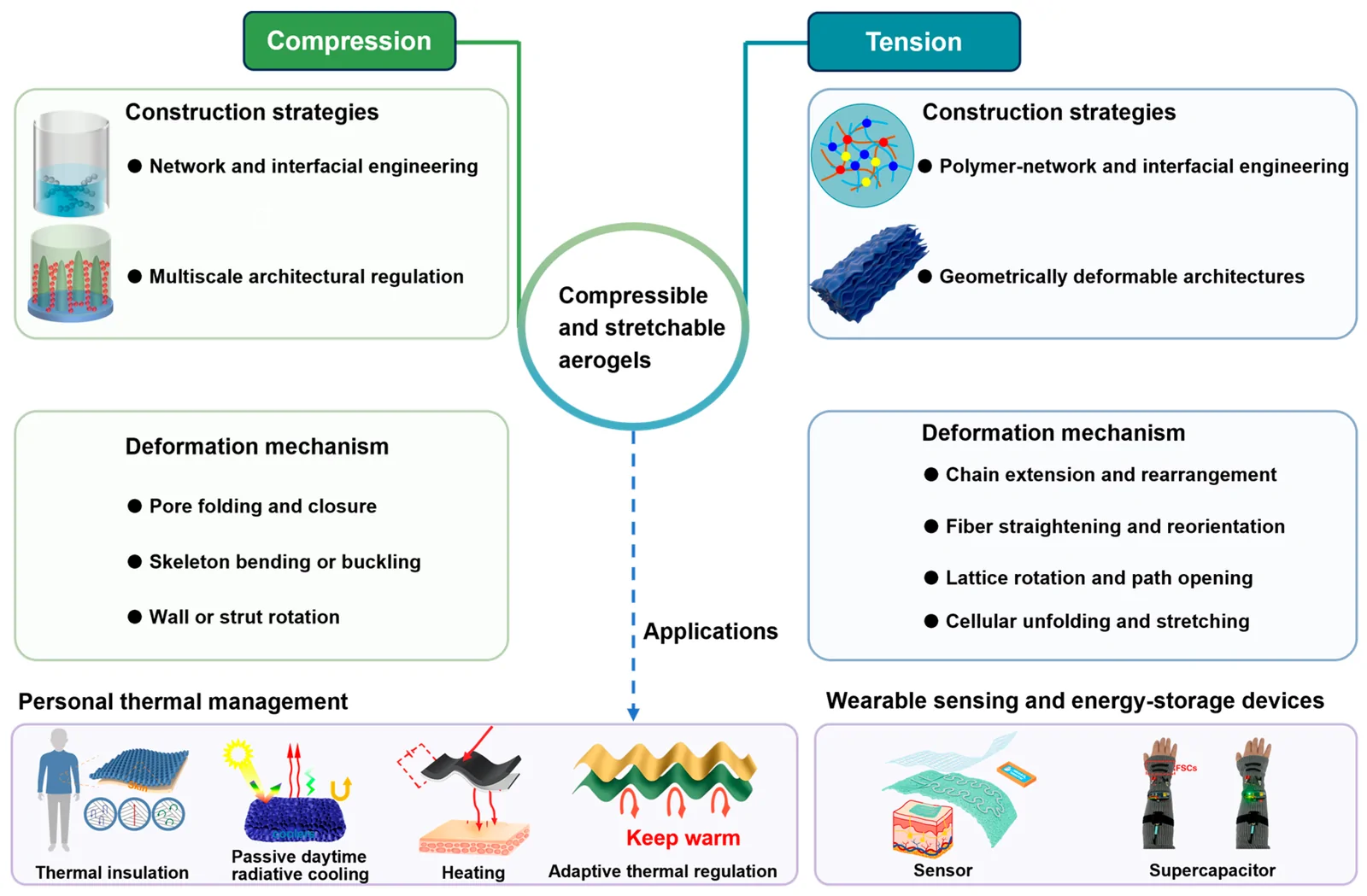
Loading-mode-specific construction strategies, deformation mechanisms, and wearable applications of compressible and stretchable aerogels.

Although previous reviews have discussed flexible aerogels from the perspectives of material composition, preparation method, mechanical performance, or specific applications, compressibility and stretchability have rarely been compared within a unified loading-mode-specific framework. This review first discusses compressible aerogels through network and interfacial engineering and multiscale architectural regulation, with emphasis on compressive deformation, elastic recovery, and fatigue resistance. Stretchable aerogels are then examined in terms of polymer-network and interfacial engineering, as well as geometrically deformable architectures. Finally, these construction principles are linked to PTM and wearable sensing and energy-storage devices, highlighting the retention of thermal, electrical, transport, and electrochemical functions during mechanical deformation (Figure 1).

## 2. Construction Strategies for Compressible Aerogels

Compressible aerogels must accommodate large deformation through reversible pore closure and skeleton bending, buckling, or rotation while avoiding brittle fracture. Their compressive performance is determined not only by the maximum strain but also by load-bearing capacity, elastic recovery, and fatigue resistance [29]. These requirements can be addressed at two coupled levels. Network and interfacial engineering imparts skeleton deformability and stabilizes load-bearing junctions, while multiscale architectural regulation provides deformation space and redistributes compressive stress.

### 2.1. Network and Interfacial Engineering

At the network level, recoverable compression can be achieved by incorporating flexible molecular segments or polymer coatings and strengthening interfacial junctions through physical, covalent, or dynamic interactions [10,22]. Across silica-, polymer-, carbon-, and MXene-based aerogels, the central objective is to balance skeleton deformability with junction stability.

#### 2.1.1. Compressible Silica-Based Aerogels

Conventional SiO_2_ aerogels consist of rigid, highly crosslinked Si-O-Si networks and defect-sensitive interparticle necks. Under compression, stress is readily concentrated at these junctions, inducing bond fracture, particle rearrangement, and irreversible pore collapse, making it hard to sustain elastic deformation [30,31]. Molecular-level tailoring of the siloxane networks is essential for converting the dominant deformation mode from brittle fracture to reversible skeleton bending and buckling. This molecular engineering generally serves two complementary purposes. The first is to decrease the effective crosslinking density and enhance the skeleton connectivity by incorporating pendant organic groups or flexible segments into the siloxane skeleton [32,33]. The second is to improve the continuity and stability of load-bearing junctions through polymeric siloxane segments or strengthened interparticle necks [34]. The introduced organic components also regulate the microstructural evolution of silica aerogels. During hydrolysis and condensation, differences in oligomer polarity affect phase separation, particle aggregation, and neck formation, thereby controlling the characteristic dimensions of the skeleton and pores [30,35,36].

**Figure 2:**
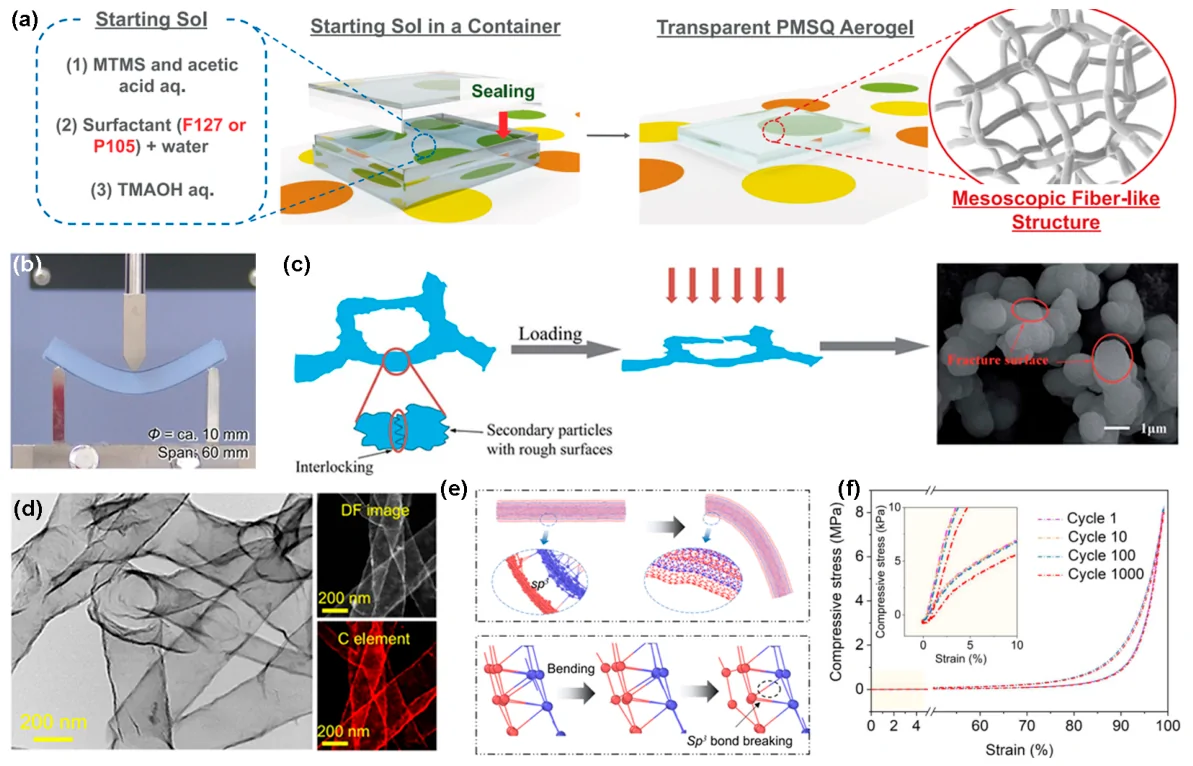
(**a**) Illustration of the preparation of poly(methylsilsesquioxane) (PMSQ) aerogels with a fibrous structure. (**b**) Photograph of PMSQ aerogels under bending. Reproduced from [37], available under terms of the CC BY 4.0 license, Copyright 2024, Ueoka R., et al. (**c**) Schematic of the structural change in the aerogel during compression. Reproduced from [38], available under terms of the CC BY 3.0 license, Copyright 2020, Zhang Y., et al. (**d**) TEM image of carbon-tube aerogel showing interconnected structure. (**e**) Molecular dynamics simulation showing the sp^2^–sp^3^ hybrid bonds and the corresponding breaking mechanism of sp^3^ bonds during bending. (**f**) Compressive stress–strain curves of a carbon-tube aerogel at 99% strain for 1000 cycles. Reproduced from [39], available under terms of the CC BY 4.0 license, Copyright 2023, Zhuang L., et al.

Polyorganosiloxane aerogels containing pendant organic groups exhibit flexibility that is synergistically governed by microstructures and network properties. By tuning the hydrophile-lipophile balance and molecular weight of nonionic surfactant, Ueoka et al. [37] induced 1D polymerization of silica nanoparticles to prepare highly transparent poly(methylsilsesquioxane) (PMSQ) aerogels (Figure 2a). An elongated fiber-like skeleton was formed, significantly improving the bendability of PMSQ aerogels (Figure 2b). This work provides a new strategy to prepare highly flexible SiO_2_ aerogels with excellent thermal insulation. The combination of dimethyldimethoxysilane and methyltriethoxysilane facilitated the transformation of the skeleton from a fragmented morphology to a coarsened and more continuous structure. The resulting thick and strong intergranular neck significantly enhance the compressive strength and mechanical stability of the framework (Figure 2c) [38]. These polyorganosiloxane aerogels exhibit high compressibility, but to maintain bending flexibility, they further require large pores and coarsened particle skeletons. The large pores provide sufficient space for reversible deformation, and the coarsened skeletons store energy through reversible buckling. Polymer-bridged networks provide another route to achieving compressibility without relying exclusively on highly enlarged pores. A series of double-cross-linked SiO_2_-based aerogels with tunable pore sizes have been developed via the classic sol–gel method [7,8,34]. For instance, nanoporous polyvinylpolydimethylsilane-based aerogels with pore sizes tunable from 20–100 nm to 2–20 μm were prepared. The network, consisting of Si-O-Si and polyethylene segments, endowed the aerogels with high compressive resilience and bendability even with nano-sized particles and pores. The methyl groups and polyethylene segments respectively reduced crosslinking density and provided ductility; thus, the stretched-side network deformed and rebounded reversibly without rigid breakage under bending. Notably, the micrometer pores allowed for twisting and curling [8]. These aerogels with homogeneous mesopores keep high bending flexibility without relying on micrometer pores.

#### 2.1.2. Polyimide (PI) and Cellulose Aerogels

Polymer-based aerogels formed through physical or chemical crosslinking generally show greater skeleton deformability than conventional inorganic aerogels. Their compressive behavior is governed by the network flexibility that in turn relies on molecular-chain mobility and the type of segmental crosslinking [40,41]. PI aerogels and cellulose aerogels represent two typical material systems in which these factors are regulated through different mechanisms. In PI aerogels, compressibility is mainly tailored by adjusting the rigidity, non-coplanarity, and mobility of the polymer backbone. In cellulose aerogels, it is primarily regulated by fibril entanglement and the physical or chemical interactions between adjacent nanofibers.

• PI aerogels: The aromatic PI nano-aerogels based on chemical crosslinking commonly exhibit superior thermal stability and mechanical toughness owing to their rigid aromatic backbones [40]. However, excessive chain rigidity and dense crosslinking restrict segmental rotation and skeleton bending. Under large compression, such skeletons are prone to stress concentration, local fracture, and irreversible rearrangement.

A classic synthesis method for crosslinked PI aerogels involves the polycondensation of dianhydrides and diamines. Firstly, polyamic acid (PAA) precursors are generated through the reaction of dianhydrides and diamines, after which the crosslinking agents are introduced, forming a percolating 3D network followed by gelation. Finally, PI nano-aerogels are acquired after imidization and drying. The flexibility of PI nano-aerogels has been improved by modulating PAA backbone structure based on flexible monomers [42,43]. For example, the flexible diamine (4,4′-oxydianiline (ODA)) was substituted for the rigid diamines in varying proportions to adjust the flexibility of PAA oligomers. The optimal combination of flexibility, moisture resistance, and thermal stability of PI aerogels was achieved at a composition containing 50% 2,2′-dimethylbenzidine (DMBZ) and 50% ODA [42]. In Shen’s work [43], different dianhydrides were chosen to react with DMBZ. Because of the strong non-coplanarity of PAA oligomers made from the flexible 4,4′-oxidiphthalic anhydride (ODPA) and DMBZ, PI aerogels with more micro- and nanometer pores exhibited excellent flexibility. In addition, the flexibility of PI aerogels is significantly increased by introducing multiple methylene units into the PI backbones, with this flexibility further increasing as the length of the diamine-derived chain segments increases [40]. For instance, PI aerogels exhibiting both superior bendability and high modulus were obtained by replacing DMBZ with 1,12-dodecanediamine in proportions of 25–75% [10].

• Cellulose aerogels: Cellulose comprises linearly linked D-glucose units via β-1,4-glycosidic bonds, with abundant hydroxyl groups forming extensive intra- and intermolecular hydrogen-bonding networks [44]. Cellulose nanocrystals (CNCs), cellulose nanofibers (CNFs), and bacterial cellulose (BC) can assemble into interconnected fibrillar networks [45]. In particular, entangled CNFs and BC fibrils with high aspect ratios provide continuous load-transfer pathways and can tolerate large compressive deformation. For instance, the CNF aerogel can withstand compressive strain exceeding 90% without breaking [46]. However, a nonelastic microstructure and strong hydrogen bonds between adjacent nanofibers can also cause irreversible interfibrillar adhesion and network densification during compression, resulting in poor elastic recovery.

Improving the resilience of cellulose aerogels requires the stabilization of the fibrillar junctions. Chen et al. [47] developed double-crosslinked cellulose aerogels using 3-aminopropyltriethoxysilane and polyvinyl alcohol (PVA), forming a robust 3D network through hydrogen and covalent bonds. The aerogels possessed enhanced compressive strength and resilience, with a compressive stress of 2.16 MPa and a maximum resilience rate of 99% under 80% strain. Incorporating highly elastic organic polymer layers is another method to reinforce the skeleton of cellulose aerogels. A smooth polysiloxane layer deposited onto the BC-based aerogel skeleton significantly improved the mechanical properties of the composite aerogels. The aerogels demonstrated a nearly complete recovery after 80% strain and a rising compressive modulus with increasing polysiloxane content (reaching 191 ± 4.6 kPa) [48]. In addition, Bhardwaj et al. [19] developed a polyurethane (PU)-reinforced CNF aerogel. The CNFs crosslinked with methyltrimethoxysilane to form a robust nanoscale 3D skeleton, while PU was uniformly deposited on the cellular walls. The PU layer absorbed deformation energy for rapid shape recovery and redistributed the applied load to prevent cellular-wall fracture under compression. Accordingly, the aerogel with the highest PU content achieved a maximum stress of 772 kPa and the fastest recovery at 80% strain.

#### 2.1.3. Carbon- and MXene-Based Aerogels

Carbon- and MXene-based aerogels have highly interconnected porous structures, high percolative electrical transport, and fast ion delivery, making them attractive for flexible electronics and energy-related devices [26,49]. One-dimensional (1D) carbon fibers and carbon nanotubes (CNTs), as well as 2D graphene and MXene nanosheets, can undergo considerable local bending without fracture. However, the intrinsic flexibility of these nanoscale building blocks does not necessarily translate into macroscopic compressive resilience of aerogels [50]. The recovery behavior of aerogels is primarily determined by interfacial slippage, adhesion, and the strength and deformability of the junctions connecting adjacent building blocks.

Graphene nanosheets with a thickness of a single carbon atom are proven to be highly elastic and robust [51]. However, graphene aerogels (GAs) have difficulty recovering from a large compressive strain due to the weak van der Waals interactions and poor slippage resistance between nanosheets. Highly elastic GAs can be obtained by strengthening the connections between nanosheets, such as maximizing π-π interactions, hybridizing with CNTs, and constructing covalent connections [49,51,52]. For instance, microwave irradiation was used to intensify π-π stacking to improve the interlayer connections of graphene oxide (GO) nanosheets within the interlocked 3D network. GAs showed enhanced stiffness and fully rebounded to their original shape after 90% compression [51]. Furthermore, Hong et al. [52] created crosslinked reduced GO (x-rGO) aerogels. PVA chains formed stable crosslinks between nanosheets and inhibited their restacking, allowing compressive stress to be transferred and dispersed through the crosslinking nodes. As a result, the x-rGO aerogels preserved a reversible strain of 60%, while conventional GAs failed at 50% strain. The chemical state of graphene sheets also affects the balance between mechanical stability and electrical transport. Oxygen-containing groups improve the dispersibility and crosslinking capability of graphene but interrupt the conjugated carbon network and reduce its conductivity [53]. Controlled reduction and carbonization enable high resilience and stable conductive pathways by eliminating oxygen-containing functional groups and defects. For instance, rGO-based aerogels were prepared by adding glucose and CNC, followed by carbonization to enhance the interlayer interactions. The aerogel could withstand an extreme strain of 99% and achieved 100% height retention after 10,000 cycles at 50% strain, with an intact conductive graphitized network [49].

MXene nanosheets possess large lateral sizes and a strong tendency toward face-to-face stacking, making it difficult to preserve an open 3D framework [6,50]. In MXene aerogels, although adjacent sheets interact through van der Waals forces and surface functional groups, these interfacial interactions generally do not form sufficiently stable junctions. Under compression, the nanosheets tend to adhere to one another, resulting in irreversible deformation [6]. Hence, the central challenge is to introduce stable interfacial interactions that stabilize the porous network without severely interrupting electrical pathways. Highly elastic polymers like thermoplastic polyurethane (TPU) and polydimethylsiloxane (PDMS) serve as binders or matrices [6], whereas waterborne polymers like CNFs [11,54] form strong hydrogen bonds with nanosheets. Conductive nanomaterials such as rGO and CNT can additionally prevent sheet restacking and establish interconnected load-transfer pathways [22]. For instance, CNFs adhered to MXene nanosheets tightly through hydrogen bonds, forming a nacre-like “brick-and-mortar” microstructure in the pore walls. The subsequent impregnation of PDMS made it possible to further realize superelasticity. CNF intercalation prevented the formation of large insulating polymer gaps between nanosheets, enabling the MXene aerogels to retain ultrahigh electrical conductivity and electromagnetic-interference shielding performance while maintaining high elasticity [54]. Superelastic MXene-based aerogels were constructed via freeze assembly with partially reduced GO and MXene. Subsequent annealing was employed to reinforce the inter-crosslinking and π-π stacking of rGO, enabling aerogels to be compressed reversibly to 95% strain [22].

CNTs can withstand large-angle and large-strain bending without structural fracture due to their exceptional mechanical strength and high elasticity [39]. CNT-based aerogels with a fibrous interconnected network demonstrate high compressive resilience through nanotube buckling, rotation, twisting, and deformation of the junctions [55]. Nevertheless, rational engineering of the junctions is essential for CNT aerogels to improve mechanical strength and elasticity. Zhuang et al. [39] designed a highly crosslinked carbon-tube aerogel containing both sp^2^- and sp^3^-hybridized structures. The introduction of sp^3^-hybridized carbon enhanced bending stability and overall strength, while interconnected junctions transferred compressive loads to adjacent carbon tubes (Figure 2d,e). These carbon tube aerogels recovered fully even at 99% compressive strain (Figure 2f) and exhibited a height loss of less than 1.5% after 1000 cycles. Similarly, 3D crosslinked CNT aerogels were prepared through a hydrothermal strategy. The junction points deformed together with carbon tubes instead of fracturing under compression. In this process, elastic energy was stored in the framework and released instantly after unloading, thus allowing the aerogels to rebound to their initial shapes [56].

As previously highlighted, network and interfacial engineering shifts brittle fracture or irreversible interunit sliding toward reversible skeleton deformation under compression. Flexible segments provide local compliance, while stable junctions preserve network connectivity, facilitate stress transfer, and promote structural recovery. Aerogels with robust elasticity can be fabricated by balancing skeleton flexibility and junction stability, whereas overly rigid cross-linking suppresses reversible bending and buckling.

### 2.2. Multiscale Architectural Regulation

Sufficient geometric space and coordinated load paths are further required for aerogels to sustain large compressive loading without localized collapse. Rational structural design enables coordinated deformation of the skeleton and pores across multiple length scales, thereby redistributing applied loads and mitigating local stress concentrations [57,58]. In this context, aerogels with compressibility, strength, and elastic recovery can be constructed by coupling these structural levels.

#### 2.2.1. Mesoscale Porous Architecture

Ice templating provides an effective route for translating the growth and spatial arrangement of ice crystals into aerogel pore architectures through the assembly of building blocks. During freezing, building blocks in an aqueous suspension are excluded from the freezing front [57]. After the ice crystals are removed, well-ordered structures (e.g., lamellar and honeycomb structures) or isotropous structures can be formed [12,57,59,60,61]. The pore diameters range from tens to hundreds of micrometers, providing sufficient space for deformation, such as pore closure and cell-wall bending or buckling. Additional structural motifs, including curved arches, interlayer bridges, and topologically interlocked interfaces, can be further incorporated into these basic architectures to redistribute stress and improve elastic recovery. The flexible building units include polymer nanofibers [62], ceramic nanofibers [13], and 2D nanosheets [63], which can be assembled into aerogels or foams with superelasticity and high strength.

Directional freeze casting establishes a temperature gradient that guides the nucleation and growth of ice crystals along a preferred direction. The suspended building blocks are expelled from the freezing front and accumulate between neighboring ice crystals, producing aligned lamellae or columnar channels after sublimation. These architectures provide well-defined load-transfer pathways and consequently exhibit pronounced mechanical anisotropy. Compression parallel to the alignment direction generally results in higher stiffness and strength. In contrast, greater compressibility and elastic recovery are achieved perpendicular to the alignment direction through wall bending and buckling. 3D honeycomb PI aerogels with orderly arranged columnar-like pores were prepared by balancing the cation-π interactions between Cu^2+^ and aromatic rings against the expansion force of ice crystals. Under compression, the ordered honeycomb structure facilitated stress transfer, while the dynamic physical interactions dissipated external energy. Consequently, this cooperation improved the compressive strength and toughness of the aerogels [64]. Zhu et al. [63] developed a gelation-constrained freeze-casting strategy for fabricating aerogels with anisotropic cellular structures. Non-covalent interactions between nanoscale building blocks formed continuous and intact cellular skeletons, which suppressed interunit slippage and promoted uniform stress transfer through skeleton buckling. This structural design significantly improved the mechanical toughness, resilience and cyclic durability of the aerogel, enabling 98% height retention after 10,000 compression cycles.

The compressive resilience of anisotropic aerogels can be further enhanced by introducing curved arches and reinforced interlayer junctions. For instance, graphene aerogels with staggered lamellar multi-arch structures were prepared via freeze casting and in situ welding. Chitosan was converted into amorphous carbon that firmly welded adjacent reduced graphene sheets under carbonization, thereby reinforcing the aerogel framework. Moreover, the staggered arches can progressively flatten via out-of-plane deformations, enabling full recovery after compression to 90% strain [29]. Similarly, nanofibrous aerogels fabricated by stacking nanofiber membranes combined flexible nanofibers, micro-arch pores, and interlayer fiber bonding. Their cooperative deformation and stabilized interfaces enabled the aerogels to recover after compression to 90% strain [65]. Inspired by the hierarchical and ordered shells of mollusks, Zhang et al. [66] prepared SiO_2_ nanofiber aerogels with a hierarchically multi-arched structure and topologically interlocked interfaces formed by bonding nanofibers and nanosheets via AlBSi. Under geometric compression, the arches accommodated large out-of-plane bending, whereas the binary bridges buckled to transfer and disperse the in-plane stress. This multiscale load-bearing mechanism endowed the aerogels with resilience at 80% compressive strain, together with bending flexibility and tensile resistance.

**Figure 3:**
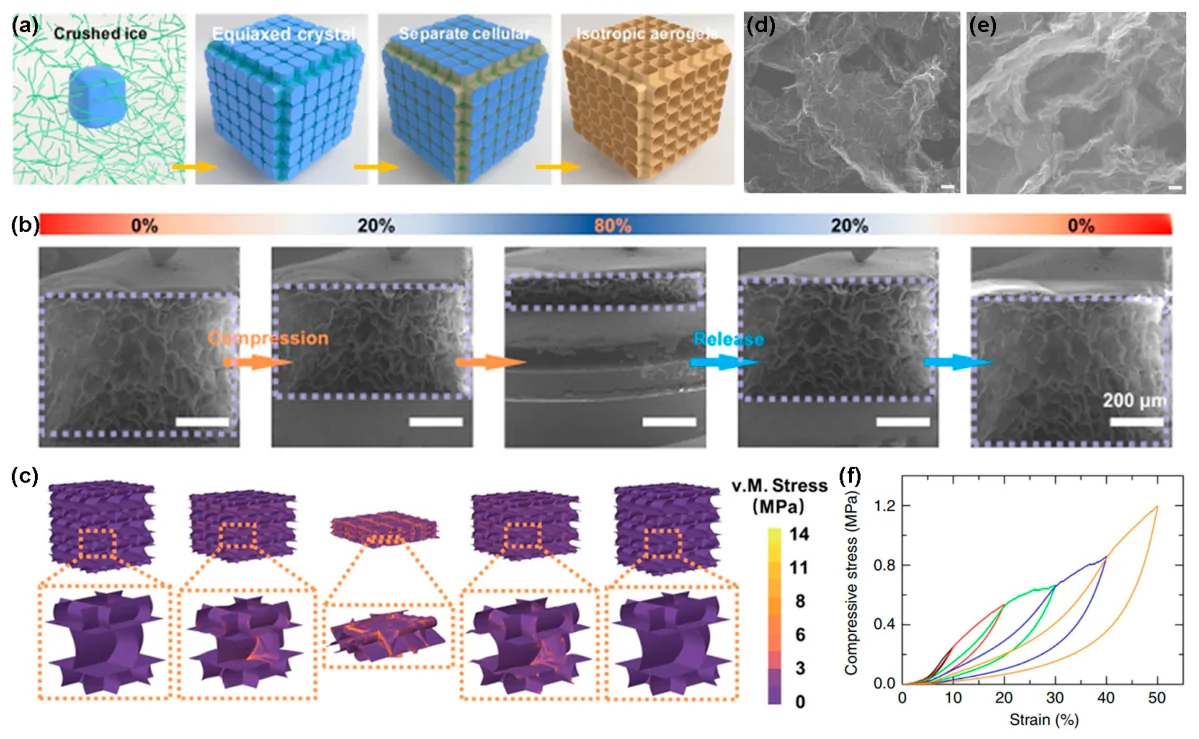
(**a**) Schematic of the preparation process of isotropous nanofiber aerogels. (**b**) SEM images and (**c**) simulation of a nonlinear finite element model showing the structural evolution of the nanofiber aerogel during compression. Reproduced from [12], available under terms of the CC BY 4.0 license, Copyright 2023, Li L., et al. SEM images of graphene aerogel microlattices (GAMs) prepared from graphene oxide (GO) ink (**d**) using resorcinol-formaldehyde (R–F) and (**e**) without R–F. (**f**) Stress–strain curves of GAMs with using the GO ink with R–F during loading–unloading cycles. Reproduced from [67], available under terms of the CC BY 4.0 license, Copyright 2015, Zhu C., et al.

Isotropic cellular aerogels exhibit comparable stress–strain responses under compression along different directions. Their interconnected and randomly oriented cell walls provide multiple load-transfer pathways, enabling stress to be distributed throughout the framework and reducing both local stress concentration and directional dependence. Li et al. [12] described an isotropic nanofiber aerogel with separate cellular structures. The process was as follows: ice crystals were produced by freezing an ultrafine nanofiber suspension and were then crushed on the surface of a rotating cryogenic drum; then the crushed crystals were mixed and re-cast with a nanofiber slurry, which induced equiaxed ice crystals to stack and integrate randomly (Figure 3a). Aerogels assembled from ultra-flexible Al_2_O_3_·SiO_2_ nanofibers exhibited superelasticity. Their thin walls underwent reversible bending and folding during compression, allowing the aerogels to fully recover to their initial shapes upon unloading (Figure 3b,c). Flexible SiO_2_ nanofiber aerogels with highly continuous interwoven cellular structures manifested compressive and bending superelasticity. This behavior originates from the reversible bending of the cell walls, enabled by the interconnections of flexible nanofibers with a length-to-diameter ratio (L/d) of 400 [13].

#### 2.2.2. Programmable Macroscopic Lattices

3D printing provides a controllable approach to construct aerogels with customized macroscopic lattices and hierarchical porous struts [58]. The printed architecture can be tailored through the geometry, dimensions, and connectivity of the lattice units, while the intrinsic nano- or microscale porous framework is retained within the printed struts [68]. In this context, the compressive performance of these aerogels depends on three related structural factors: the topology of the macroscopic lattice, the porous structure of the struts, and the coupling between these two structural levels.

Lattice topology governs the geometric freedom for macroscopic deformation and the dominant pathways of load transfer. Open lattices, such as woodpile and honeycomb architectures, generally accommodate large compressive strains through strut bending, wall rotation, hinge deformation, and progressive closure of the lattice openings. These deformation modes reduce local stress concentration and are favorable for high compressibility and elastic recovery. Ca^2+^-crosslinked GO inks were used to construct woodpile microlattices, whose pore sizes ranged from hundreds of nanometers to tens of micrometers. The resulting macroscopic lattice geometry endowed the aerogels with structural stability and enabled elastic deformation, allowing the aerogels to recover effectively after compression to 80% strain [69]. Gui et al. [70] fabricated periodic honeycomb-like PI aerogels using freeze-casting-assisted extrusion printing. A stable lattice was formed by the interconnection of nodes in hexagonal units. When the aerogels were compressed, the hinges at the junctions deformed to absorb mechanical energy and transmit and redistribute stress throughout the lattice. Thus, high compressive strength was achieved. Introducing triangulated units provides more continuous load-bearing paths and generally increases stiffness and strength. SiC-nanowire/silica aerogels were printed with square, hexagonal, and triangular lattices, showing topology-dependent mechanical behavior. Among them, triangular-lattice aerogels exhibited the highest Young’s modulus [71].

The bending and buckling of the porous structure within the aerogel struts govern their capability to store elastic energy and resist irreversible fracture or densification. Open cellular and fibrous skeletons generally allow greater local deformability, whereas dense or excessively crosslinked skeletons increase stiffness. Zhu et al. [67] reported periodic graphene aerogel microlattices (GAMs). GO inks with shear-thinning behavior were first obtained via SiO_2_ thickening and resorcinol-formaldehyde (R–F) crosslinking. Then aerogels with cubic-like lattices and tunable microstructures were constructed (Figure 3d,e). The high resilience of GAMs relied on a less crosslinked and more open framework (Figure 3f), while the macroscopic architectures provided superior mechanical strength. Jiang et al. [72] developed an all-cellulose gel-like ink to construct a super-strong aerogel. Strong hydrogen bonding between cellulose chains endowed the aerogels with a high compressive modulus of 16.6 MPa, while disruption of these interactions after water absorption decreased the modulus to 170 kPa, thus demonstrating superior flexibility.

The compressive performance is markedly enhanced when the designs of macroscopic lattices and porous skeletons are combined. In such hierarchical architectures, the lattice provides geometric freedom for large-scale deformation and redistributes the applied load, whereas the porous framework accommodates local strain. For highly elastic aerogel microlattices, pre-reduced GO inks were employed to fabricate graphene-based materials with hierarchical structures. Under compressive loads, the macroscopic openings of the microlattice closed, mitigating local strains. Meanwhile, the ordered cellular microstructures with thicker walls and strong bonding buckled. Both mechanisms contributed to compressive resilience at a strain of up to 95% and enhanced modulus [58]. Mechanically robust, ultraelastic PU foams were fabricated via sequential processes of direct ink writing, acid etching and phase inversion. The large, well-organized honeycomb-like pores and macroscopic arrays, provided more space for the PU foam to deform through buckling and bending of the cell walls or deformation of the filaments. Thus, extraordinary elasticity and robustness in PU foams were achieved [73]. To facilitate comparison of these coupled effects, representative additively manufactured aerogel and foam lattices are summarized in Table 1 in terms of material composition, macroscopic architecture, dominant deformation characteristics, and mechanical performance.

Overall, multiscale architectural regulation provides aerogels with geometric freedom for deformation and governs load transfer under compression. Cellular-structured aerogels accommodate local strain through wall bending and pore closure. Moreover, programmable aerogel lattices can redistribute loads through the coordinated deformation of struts and nodes, while the topology regulates their macroscopic stiffness and strength. Their effective coupling is essential for simultaneously achieving large compressibility, recovery, and load-bearing capacity.

## 3. Construction Strategies for Stretchable Aerogels

Stretchable aerogels place stringent demands on network continuity because tensile loading generates stress concentrations at network junctions and interfaces, thereby promoting crack propagation and network disconnection. Large elongation must be achieved without disrupting continuous load-transfer pathways or causing irreversible interfacial separation [80]. These requirements can be addressed by constructing extensible polymer or hybrid networks and introducing geometric redundancy.

### 3.1. Polymer-Network and Interfacial Engineering

Polymer-containing networks provide the molecular mobility required for tensile deformation, but the stretchability of aerogels also depends on their network junctions and interfaces that can preserve network continuity under load [16]. In aerogels with continuous polymer networks, elongation is mainly achieved through molecular chain rotation, extension, disentanglement, and deformation of the network junctions [81]. In polymer-integrated aerogels, a polymer is combined with an inorganic component and serves as a flexible bridge [82], interfacial binder [16], or secondary reinforcing network [83].

#### 3.1.1. Constructing Intrinsically Stretchable Polymer Networks

Polymers with intrinsically flexible and mobile molecular chains can directly form stretchable porous networks. For instance, a PU aerogel based on a triisocyanate precursor exhibited an elongation at break exceeding 120% [81]. Chen et al. [14] constructed a PI aerogel featuring an intrinsically branched and highly connected framework. Increasing the proportion of the flexible dianhydride enhanced the rotational freedom and mobility of the polymer sub-chains, while fiber bundling and junction formation generated a branched fiber-like network. This multiscale structural design enabled the PI aerogels to achieve an elongation at break over 30% and a high tensile strength of 17.633 MPa.

**Figure 4:**
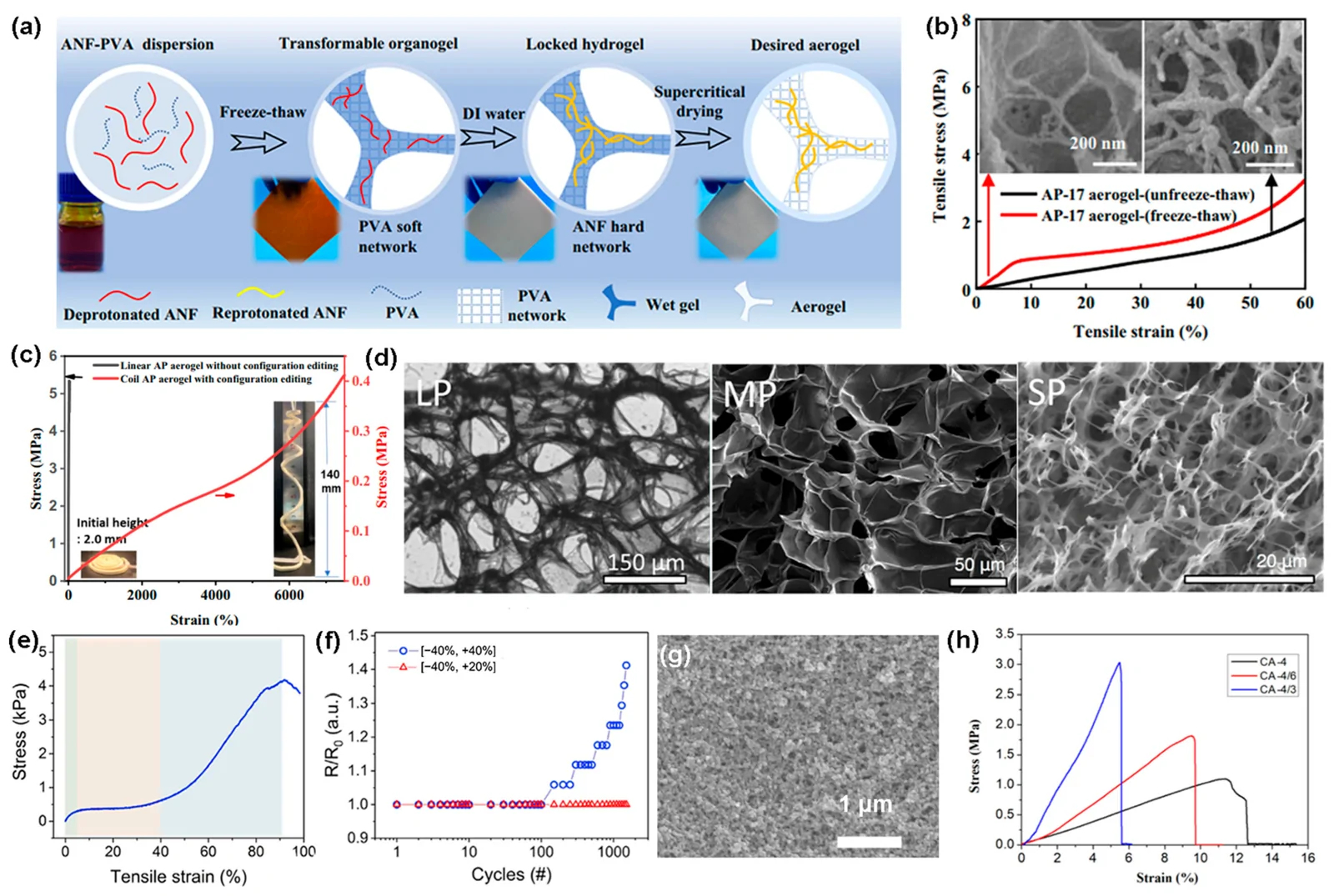
(**a**) Schematic of the evolution of the ANF-PVA gel-network during twice coagulation. (**b**) Tensile stress–strain curves and SEM images (insets) of aerogels with and without a freeze-thawing process. (**c**) Tensile stress–strain curves of the coil aerogel and aerogel without configuration editing. Reproduced from [15], available under terms of the CC BY 4.0 license, Copyright 2023, Li. L et al. (**d**) SEM images of PEDOT aerogels with different pore sizes (denoted LP for large pore, MP for medium pore, and SP for small pore). (**e**) Tensile stress–strain curve of the LP aerogel. (**f**) Cycling resistance variation in the LP aerogel cycled between different strain ranges. Reproduced from [85], available under terms of the CC BY NC-ND 4.0 license, Copyright 2019, Chen G., et al. (**g**) SEM image of silica–cellulose aerogel prepared with 6.8 mL of silica precursor (CA-4). (**h**) Tensile stress–strain curves of CA-4 and after compression treatment in the wet gel state. Reproduced from [91], available under terms of the CC BY 4.0 license, Copyright 2020, Sai H., et al.

Stable crosslinking within the polymer network is further introduced to combine large elongation with structural stability. Polydopamine (PDA)-anchored CNF was used to crosslink with PVA and imine species to construct polymer aerogels. Boronate and imine covalent bonds constructed an adjustable flexible network, while the PDA-anchored CNFs served as a reinforcing framework. The combination of dynamic chemical bonds and rigid nanofibers enabled efficient stress transfer, endowing the aerogels with high tensile strength [84]. Li et al. [15] proposed a twice-coagulation strategy. As illustrated in Figure 4a, PVA was polymerized to form a flexible gel network, which offered the structural foundation in the first coagulation process. During the second coagulation, an aramid nanofiber (ANF)-PVA configuration-locking was achieved via π–π interactions between ANFs and hydrogen bonding between ANF amide groups and PVA hydroxyl groups. A continuous wall-like framework was formed within the aerogel. The tensile strength of the aerogel was markedly improved with increasing cross-linking density (Figure 4b). Furthermore, a highly elastic gel editing and fixing process can produce a coil structure with an elongation of 7000%, breaking through the mechanical constraints (Figure 4c).

As a class of stretchable aerogels, conductive polymer aerogels can be prepared via some special polymers and applied in wearable electronic devices [85,86]. Chen et al. [85] created stretchable aerogels with a tunable pore size ranging from 5 μm to 100 μm based on poly(3,4-ethylenedioxythiophene): polystyrene sulfonate (PEDOT:PSS) (Figure 4d). The combination of highly stretchable PEDOT, a chemically crosslinked 3D network, and micrometer-scale cellular sizes provided the aerogels with a tensile strain of 90% (Figure 4e). The aerogels maintained stable resistance change over a strain range of −40% to 40% under tensile cycling (Figure 4f). Within the plateau regime of the stress–strain curve, stretching was primarily accommodated by pore torsion and elongation. The PU-reinforced PEDOT:PSS aerogel film, prepared using a uniaxial pre-stretching strategy, exhibited a high elongation of 100–200% [87].

#### 3.1.2. Polymer-Integrated Hybrid Networks

In polymer-integrated hybrid aerogels, polymers are structurally coupled with a distinct secondary phase, such as nanosheets, nanowires, and particles, or with an inorganic network. The polymer may form a continuous matrix or conformal coating that integrates the secondary phase, bridge adjacent building blocks, or act as flexible segments within an organic–inorganic network. Their tensile response is governed by both polymer deformation and stress transfer across heterogeneous interfaces.

When the polymer forms a continuous matrix or coating, it accommodates most of the tensile deformation while stabilizing a functional framework. PDMS was used to infiltrate hierarchically porous silver nanowire (AgNW) aerogels, producing a continuous compliant matrix around the conductive network. The hybrid aerogel achieved a high electrical conductivity of 65.7 S/cm, with a large tensile strain of 130% [88]. Similarly, TPU-reinforced MXene-based foams with a continuous, interconnected 3D network were designed. Controlled coagulation of TPU within the BC-bridged MXene framework formed a neuro-like network, which accommodated reversible deformation and enhanced stretchability. Consequently, the foams retained high conductivity over a wide recoverable strain range of −80% to 80% [89]. In a hydrophobic silica aerogel particle (SSAP)/TPU composite, the porous TPU matrix constituted the primary extensible skeleton, endowing the aerogel with stretchability. Moreover, dispersed SSAPs hindered crack propagation, enhancing the tensile strength. Accordingly, the composite exhibited an elongation at break approaching 500% at an SSAP content of 5 wt% [83].

In other hybrid networks, polymer chains can crosslink with the building blocks to form stable junctions that facilitate the retention of network integrity. However, overly rigid or dense crosslinking constrains segmental mobility and thus increases stiffness at the expense of elongation. Zheng et al. [16] developed a self-foaming strategy to prepare polyurea/MXene aerogels involving monomer polymerization, MXene dip-coating, and GO-assisted multiple crosslinking. The soft segments of polyurea chains endowed the aerogel with a high elongation of over 160%, whereas the hard segments formed hydrogen-bonded domains that enhanced the network strength. Meanwhile, hydrogen bonds formed by GO, polyurea and MXene firmly anchored the MXene sheets to the polymer cell wall, forming a continuous conductive network and mechanically reinforcing phase. This synergistic interplay increased the tensile strength of the aerogels to 2.72 MPa while maintaining an elongation at break of 85.9%. An interfacial enhancement strategy was proposed, in which PI macromolecular chains bridged adjacent MXene nanosheets and formed a robust sandwich-structured framework. The stretchable microfolds and cell walls of the aerogels contributed to reversible stretchability (20% strain) and cyclic tensile deformation resistance [90].

Polymer-based hybrid networks combine flexible organic components with inorganic skeletons through covalent or interfacial interactions. The organic phase accommodates tensile deformation, whereas the inorganic framework enhances structural stability and load transfer. Silica sponges, prepared via the hydrolysis-condensation of methylsiloxanes, possessed a micrometer-scale open-cell structure, exhibiting a tensile strain of approximately 50%. Their stretchability arose from the cooperation of deformable pores, coarsened skeleton junctions, and organic groups within the siloxane framework [82]. Silica–cellulose composite aerogels were constructed with a nanoscale interpenetrating network (IPN) consisting of a silica gel skeleton and a BC nanofiber framework. The BC nanofibers were intertwined with silica skeletons at the nanoscale, forming an integrated IPN structure (Figure 4g). The tough BC nanofibers served the primary load-bearing phase, effectively distributing and transferring external forces. Meanwhile, the rigid silica skeleton restricted the microscopic displacement and sliding of BC nanofibers during tensile deformation, thereby increasing the tensile modulus of the composite material (Figure 4h) [91].

In summary, polymer-network and interfacial engineering enables stretchable aerogels to maintain tensile continuity through molecular extensibility and controlled interfacial stress transfer. Continuous polymer networks favor large elongation, whereas hybrid networks incorporate reinforcing phases to enhance strength and functional stability. The central challenge is to provide sufficient chain and junction mobility for deformation while maintaining interfacial integrity and resistance to failure.

### 3.2. Geometrically Deformable Networks and Architectures

Geometrically deformable aerogels can be constructed by introducing recoverable geometric features across multiple length scales, including crimped fibrous networks [80], wrinkled or folded cellular skeletons [92], and curved or programmable macroscopic architectures. Specifically, curly and flexible nanofibers or nanowires can entangle to form crimped networks, whereas crosslinked nanosheets can be transformed into wrinkled or folded structures through self-assembly and subsequent processing. At the macroscopic scale, coiling, folding, or patterned fabrication can further encode curved deformation pathways into the aerogel architecture. These aerogels incorporate excess contour length or rotational freedom into their architectures, allowing tensile strain to be accommodated through geometry-mediated deformation rather than direct stretching of the constituent skeleton.

#### 3.2.1. Crimped and Entangled Fibrous Networks

Randomly interwoven flexible nanofibers with high aspect ratios and continuity have been explored to construct fiber-structured aerogels [80,93]. Their tensile behavior is jointly governed by fiber curvature, intrinsic fiber toughness, and the connectivity and stability of network junctions. During stretching, buckled fibers progressively straighten and reorient, while robust nanofibers and reliable entanglement/connection maintain load transfer and network integrity. In this context, these aerogels show tensile flexibility and enhanced resistance to fracture.

**Figure 5:**
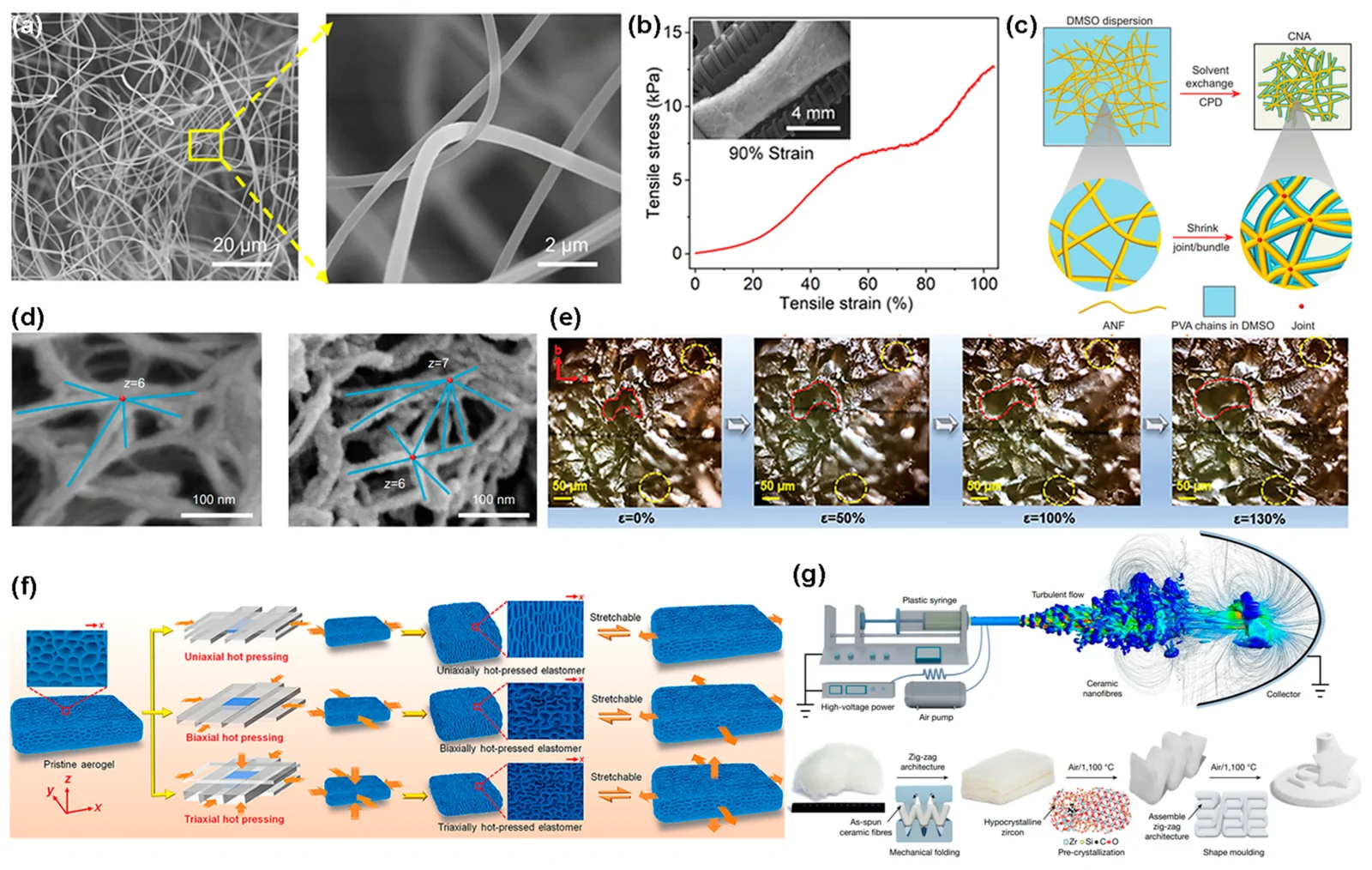
(**a**) SEM images of a ceramic nanofibrous aerogel with knitted crimped-nanofiber structure. (**b**) The tensile stress–strain curves and photo of mullite nanofibrous aerogels. Reproduced from [17], available under terms of the CC BY 4.0 license, Copyright 2022, Cheng X., et al. (**c**) Sketch of the assembly process of the ANF-based aerogels. (**d**) SEM images of ANF-based aerogels showing high-connectivity nodes. Reproduced from [95], available under terms of the CC BY 4.0 license, Copyright 2022, He H., et al. (**e**) Preparation of stretchable rGO/polymer elastomers via hot-pressing strategies. (**f**) In situ optical microscope images of the uniaxially hot-pressed rGO/polymer aerogel during stretching. Reproduced from [96], available under terms of the CC BY 4.0 license, Copyright 2024, Zhang X., et al. (**g**) Illustration of the fabrication process of ceramic aerogels. Reproduced from [97], available under terms of the CC BY 4.0 license, Copyright 2022, Guo J., et al.

Ultrahigh and robust stretchability can be achieved by designing a crimped and entangled structure. Cheng et al. [17] prepared mullite nanofibrous aerogels with an interwoven, crimped nanofiber structure via sol–gel electrospinning (Figure 5a). When the aerogels were subjected to large tension, the crimped and tangled flexible nanofibers turned to decline, deform, orientate, and interlock. Meanwhile, the junctions redistributed stress across the entangled network. Moreover, the porous structure provided sufficient space for fiber deflection and rearrangement. These features enabled the aerogels to withstand a tensile strain of 100% (Figure 5b). A two-component off-axial electrospinning method was developed to construct an ultra-stretchable aerogel. An appropriate Zr-Si ratio and hypocrystalline state endowed individual nanofibers with toughness, while the highly buckled structure converted lateral deformation into axial elongation. The resulting aerogels reached a maximum stretchability as high as 150% and recovered after stretching to 80% strain [93].

SiC-SiO_x_ aerogels, constructed from bicrystal nanowires by high-temperature vapor deposition, exhibited both high compressibility and stretchability. Multiple mechanisms worked together, contributing to an elongation at break of 20%. These mechanisms concretely show that the curly nanowires and bundles reoriented parallel to the tensile direction, enabling them to withstand a larger tensile load. Meanwhile, the resulting compressive stress stemming from buckling deformation of perpendicular nanowires offsets tensile stress [94]. In another design, ethanol-induced aggregation and drying generated wrinkled layers in SiC-SiO_x_ nanowire aerogel paper. The tensile deformation of aerogel paper was accommodated through the unfolding of soft wrinkles and movement of nanowires, while the intensive interactions and planar constraint within laminated layers suppressed excessive sliding and improved tensile strength [80].

Polymer nanofibers with structural connectivity can be explored to fabricate aerogels or foams with a stretchable 3D microfibrillar network. Their tensile properties are related to fiber-fiber interactions and the intrinsic properties of the polymers. ANFs were assembled into highly connected networks, where PVA molecular chains interacted with ANFs, leading to the entanglement and crosslinking of ANF bundles (Figure 5c). A computational model confirmed that the elongation of the nanofiber aerogels was governed by the deformation, breakage, and bending of PVA at nodal points. Increasing the connectivity of welded nodal points produced a high tensile modulus (Figure 5d), limiting the elongation at break to 22.25–27.4% [95]. A fluffy sponge with curly nanofibers and semi-interpenetrating polymer networks (semi-IPN) was fabricated via electrospinning and thermal crosslinking (initiated by isocyanate-based polymer and PU). The curly fibers provided geometric extensibility, while the semi-interpenetrating network preserved connectivity and dissipated tensile energy. Accordingly, the sponge withstood a tensile strain exceeding 40% and exhibited negligible plastic deformation after 1000 tensile cycles at 30% strain [98].

#### 3.2.2. Pre-Deformed Cellular Structures

Pre-deformed cellular structures contain excess contour length within crimped, folded, or wrinkled pore walls before tensile loading. During subsequent stretching, this contour length can be released through the unfolding and stretching of the cell walls. Large elongation is primarily enabled by geometric redundancy, preventing the constituent skeleton from directly bearing the full macroscopic tensile strain. Lamellar, cellular, and honeycomb-like structures may distribute and transfer stress, thereby improving structural integrity and facilitating the generation of such a geometric architecture [96]. Specifically, this architecture can be introduced either by compressing and fixing a cellular aerogel or by forming the porous aerogel on a pre-stretched substrate [87,96].

When a highly compressible aerogel is annealed in a compressed state through a hot-compression strategy, its cell walls undergo folding, crimping, and structural fixation, forming folded or wrinkled structures. During subsequent stretching, these geometric features can progressively unfold, providing excess deformation capacity and thereby enabling ultrahigh stretchability. A series of ultra-stretchable aerogels with a highly crimped, folded cellular structure was developed [92,96,99]. Zhang et al. [96] created highly stretchable porous rGO/polymer elastomers (Figure 5e). During the hot-pressing process, folded pore walls and new weak contact points were produced, while the polymer chains underwent deformation and rearrangement. After cooling, both the pore wall geometry and the newly established contacts were fixed. The uniaxially hot-pressed porous elastomers exhibited an elongation at break of 1250% thanks to the unfolding of folded pore walls and the high deformability of polymers (Figure 5f). Biaxial hot-pressing constructed a re-entrant porous structure in elastomers, which led to a negative Poisson’s ratio. A crimped MXene-based aerogel was prepared through PVA-assisted assembly followed by uniaxially hot pressing. In situ morphological observation further confirmed that the pore walls of the aerogels gradually unfolded and stretched, while the pore dimensions changed primarily along the stretching directions. This geometry-mediated deformation enabled the aerogels to achieve an ultrahigh elongation of up to 427% [92].

Pre-stretching strategies are also employed to fabricate conductive aerogel films based on an elastomeric substrate. After gelation and drying, release of the substrate imposes in-plane compression on the attached porous film, producing wrinkles and crimped cellular walls. For instance, uniaxial and biaxial pre-stretching strategies combined with crosslinking and template methods were utilized to fabricate several types of semiconducting polymer-based aerogels. The sol–gel and drying processes took place on PU substrates pre-stretched to 50–200%, after which aerogel films with crimped and folded structures were formed. Notably, PEDOT-UP200 (a PEDOT:PSS aerogel film with a pre-stretching ratio of 200%) displayed a maximum tensile strain of 200% [87]. Yang et al. [23] developed stretchable aerogel films by depositing semiconducting polymers on the porous gel skeleton. The crosslinked gel network formed by PVA and GO provided flexibility, and the skeleton with a folded and crimped structure further unfolded and stretched. Accordingly, the aerogel film with 50% uniaxial pre-stretching showed a tensile strain of 30–40%.

To facilitate a direct comparison of the construction strategies discussed above, Table 2 summarizes representative stretchable aerogels in terms of their material composition, construction methods, structure, tensile performance, and cyclic durability.

#### 3.2.3. Programmable Macroscopic Architectures

Macroscopic architectures introduce geometric extensibility at a scale larger than the intrinsic pores of the aerogel. Numerous special macroscopic architectures, such as lattices, serpentines, springs, and wrinkles, possess high deformability and elasticity. Additionally, 3D printing has been explored to design these architectures in a programmable way. When combined with highly elastic microstructures and suitable aerogel composition, the resulting architectures enable ultrahigh stretchability [99].

Periodic lattices accommodate tensile deformation through changes in the angles and relative positions of interconnected struts. Re-entrant or hinged units rotate and open around their nodes, converting local bending and rotation into macroscopic elongation. In a printed Kevlar nanofibrous aerogel, the elongation was governed by the angular changes in the interconnected struts and mesopores within the struts. Specifically, the re-entrant honeycomb unit-cell array produced a negative Poisson’s ratio, whose value was determined by the periodic macroscopic architectures rather than the intrinsic elasticity of the materials. Printed carbon aerogel lattices similarly combined node rotation with deformation of their porous struts, achieving a 200% elongation with elastic recovery. The mechanism can be inferred as follows: the rotation of millimetric hinged struts, the deformation of units enclosed by CNT-interconnected graphene laminates, and the stretching of folded cell walls worked synergistically [18].

Curved continuous paths provide larger geometric redundancy than conventional lattices. Graphene aerogels patterned into spring and serpentine architectures using a laser-engraving strategy presented elongations of approximately 5400% and 1200%, respectively. In contrast, monolithic GAs with the same microstructures showed a minor elongation at break of 6%. The exceptionally high elongations originate primarily from progressive opening and straightening of the macroscopic path [100]. Similarly, Guo et al. [97] reported nanofibrous aerogels with zig-zag architectures, fabricated through electrospinning with a coaxial air-blowing configuration, mechanical folding, and subsequent high-temperature treatment and crosslinking (Figure 5g). Under tension, the zigzag units progressively opened, enabling a large elongation of 40%. Meanwhile, strong hypocrystalline zircon nanofibers and their entangled junctions preserved the integrity of the path. More importantly, a near-zero Poisson’s ratio and negative thermal expansion coefficient were achieved due to the zigzag architecture, enabling thermomechanical stability.

### 3.3. Hierarchical Organization of Stretchable Aerogel Fibers

The network- and geometry-based strategies discussed above can be further translated into 1D aerogel fibers for textile integration [5]. Aerogel fibers combine an internally porous structure with a continuous macroscopic filament, allowing them to be stretched, twisted, bundled, and woven into conformable textiles [101]. For PTM, these fibers offer sufficient stretchability and tensile strength in addition to superior thermal insulation. Their tensile performance can be tailored by reinforcing the fibrous framework and spatially organizing regions with distinct mechanical roles.

High axial load-bearing capacity can be achieved by strengthening the continuous network within individual fibers or by assembling multiple fibers to promote load sharing. Jiang et al. [102] fabricated cellulose aerogel fibers via continuous coagulation spinning followed by modification with diisocyanate (MDI). The flexible cellulose molecular chains were locked by an appropriate amount of MDI crosslinking agent (1.5%), increasing the tensile modulus to 735 MPa. This result mainly demonstrates the enhancement of axial load-bearing capacity. The development of high-tensile-strength biomass aerogel fibers provides a reliable reference for wearable PTM. In addition, PI aerogel fibers were twisted into fiber bundles with different fiber numbers and twisting levels, providing a structural strategy for improving tensile properties. Fiber interlocking, binding strength, and friction between fibers collectively promoted load transfer and progressive failure, thereby delaying catastrophic fracture and increasing the tensile strength. Fiber bundles with the optimal twisting level and fiber number showed an elongation at break of 66.5% and high robustness [103].

Heterostructured aerogel fibers have been developed to balance tensile strength, stretchability, and porous functionality by assigning different mechanical roles to spatially separated regions. Core–shell fibers (EAFs) were fabricated by freeze spinning, followed by encapsulation of the lamellar aerogel fiber with stretchable TPU. The aerogel core preserved low density and thermal insulation ability, whereas the dense TPU shell maintained structural continuity and accommodated large tensile deformation. Increasing the TPU thickness to ~80 μm raised the elongation of EAFs to 1000%. The textile woven from EAFs retained stable thermal insulation properties after 10,000 stretching cycles at 100% strain [101]. Zhu et al. [104] developed core–shell PI fibers, in which long PI fibers (core layer) were tightly encapsulated by a porous crosslinked PI aerogel (shell layer). The high-strength PI fibers bore the primary stress when tensile loading was applied to the composite aerogel fibers. Accordingly, high tensile strength and large tensile strain could be achieved by adjusting the proportion of long PI fibers. Similarly, a flow-assisted dynamic dual-cross-linking strategy based on a microfluidic chip was proposed to fabricate cellulose sponge-aerogel fibers. These fibers possessed a mechanically robust sponge layer and a highly porous aerogel. The thickness ratio between the sponge and aerogel layers, together with the total fiber diameter, determined the tensile strength and stretchability. The optimal fibers reached a tensile strength of 71.83 MPa, significantly higher than that of the corresponding sponge and aerogel fibers. Moreover, the woven textiles tolerated repeated folding and bending [105].

Both compressible and stretchable aerogels share network- and architecture-based construction principles. Nevertheless, their specific structural requirements differ substantially because of the distinct loading modes of compression and tension. Table 3 summarizes the loading-specific deformation mechanisms, construction strategies, design requirements, and wearable relevance of compressible and stretchable aerogels. Under wearable compression, compressible aerogels accommodate contact pressure and repeated local deformation through reversible pore closure and skeleton reconfiguration. By contrast, stretchable aerogels maintain structural continuity during body motion through molecular extension, interfacial stress redistribution, and geometry-mediated elongation. These loading-specific mechanical responses, in turn, govern the stability of porous structures and thermal, electrical, and transport pathways during deformation. Despite these differences, both design routes ultimately aim to preserve structure-dependent functions during repeated deformation, thereby providing the mechanical basis for the wearable applications discussed below.

## 4. Wearable Applications of Compressible and Stretchable Aerogels

Wearable applications place simultaneous demands on mechanical compliance and functional stability. The compressive and tensile design strategies discussed above enable aerogels to accommodate contact pressure and body motion while preserving porous, optical, conductive, and electrochemical pathways required for device operation. Depending on the incorporated functional components and transport pathways, mechanically compliant aerogels can serve in two major categories of wearable applications: PTM and sensing and energy-storage devices.

### 4.1. Personal Thermal Management

PTM regulates heat exchange within the local microenvironment surrounding the human body [106]. Flexible aerogels combine porous thermal resistance with tunable optical, electrical, and heat-storage functions while maintaining conformal contact during body motion [107,108,109]. Their applications include thermal insulation, passive daytime radiative cooling, photothermal and electrothermal heating, and adaptive or switchable thermal regulation.

#### 4.1.1. Thermal Insulation

Wearable thermal insulation primarily relies on suppressing heat loss through conduction, convection, and radiation. The nanoporous aerogel skeleton reduces solid and gaseous heat conduction, while highly reflective components can further limit the escape of body-emitted mid-infrared (MIR) radiation. Aerogel fibers are particularly attractive because they preserve these porous characteristics within continuous filaments that can be woven or knitted into textiles. After conductive heat transfer is minimized, infrared-reflective components can be further introduced to reduce radiative heat loss from the body. Continuous aerogel fibers provide the structural basis for lightweight insulating textiles. Liu et al. [110] reported a Kevlar aerogel fiber by a wet-spinning method. A single fiber of this type exhibited an elongation at break of 35.2% and a tensile strength of 3.3 MPa, along with a thermal conductivity of 0.027 W·m^−1^·K^−1^. Its interconnected porous structure suppressed heat conduction and convection, whereas the tough fiber feature enabled weaving into textiles. Hence, the textile possessed superior thermal insulation performance in both contact tests at −196 and 350 °C and room-temperature tests conducted on human skin surfaces.

**Figure 6:**
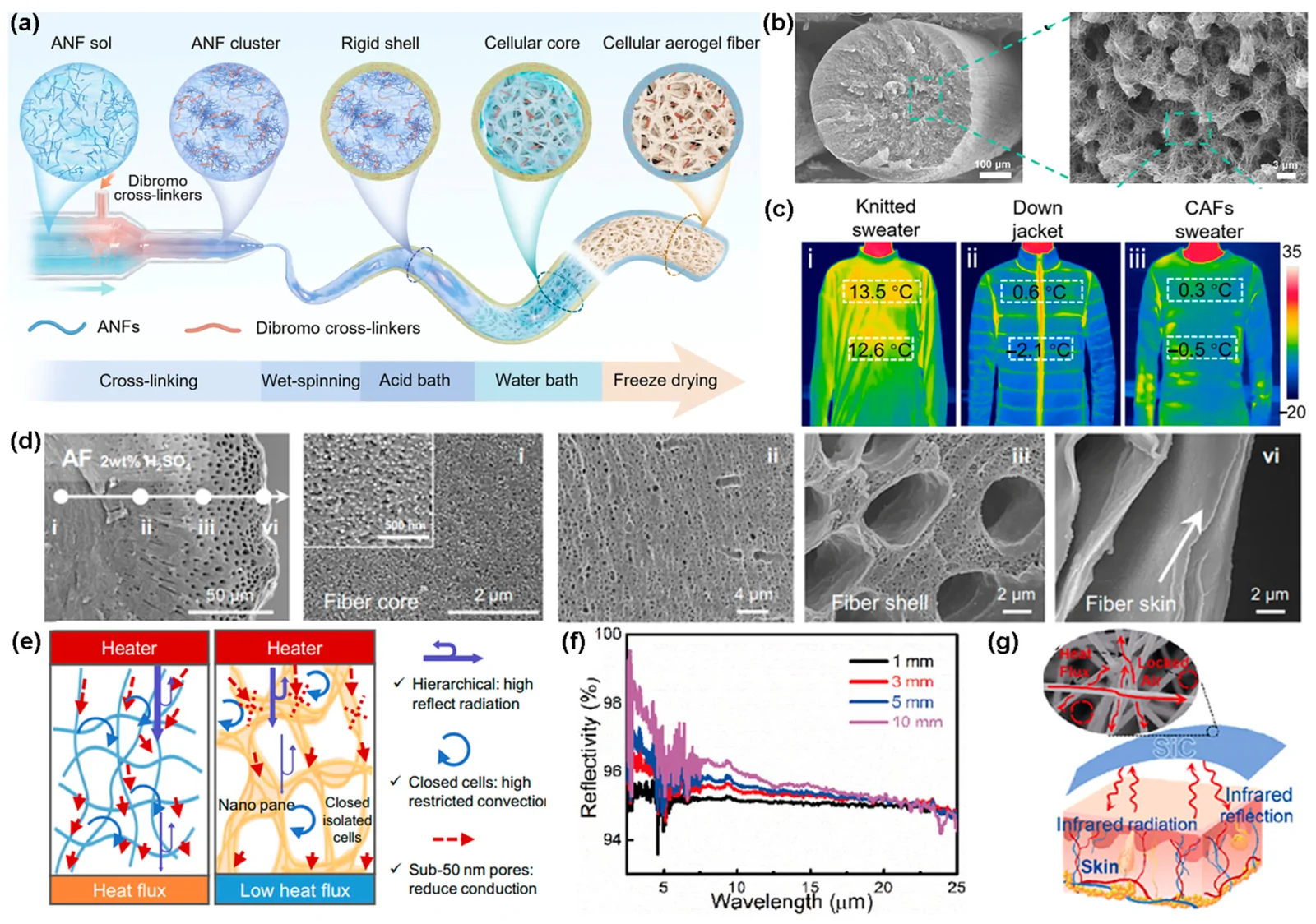
(**a**) Illustration of a wet-spinning process to produce CAFs. (**b**) SEM images of cross section and magnified core region of a CAF. (**c**) Infrared images of a volunteer wearing a commercial sweater, a down jacket, or CAF fabrics measured at −20 °C. Reproduced from [111], available under terms of the CC BY-NC-ND 4.0 license, Copyright 2026, Hu Y., et al. (**d**) SEM images of HANF-AFs with a hierarchically porous structure in a 2 wt% H_2_SO_4_ coagulation bath. (**e**) Schematic illustration of the thermal-transfer suppression of aerogel fibers of HANF-AFs. Reproduced from [112], available under terms of the CC BY-NC-ND 4.0 license, Copyright 2026, Xiao G., et al. (**f**) Infrared reflectivity of the SiC nanofiber aerogels at different thicknesses. (**g**) Schematic of infrared radiation and reflection between skin and the SiC nanofiber aerogels. Reproduced from [113], available under terms of the CC BY 4.0 license, Copyright 2022, Song L., et al.

Heterogeneous aerogel fibers can further improve mechanical reliability while preserving the thermal insulation performance. Bionic honeycomb-structured aerogel fibers (CAFs) were prepared by dual-coagulation-bath spinning (Figure 6a). The highly oriented and crystalline sheath provided a high tensile strength of 74.6 MPa. In contrast, the honeycomb architecture, featuring multiscale pores and nanoporous cell walls (Figure 6b), reduced deformation resistance and resulted in extremely low bending and compressive moduli. Meanwhile, the multiscale porous sheath and core cooperatively suppressed heat transfer, enabling effective thermal insulation. A 0.9 mm-thick sweater woven from these aerogel fibers provided a warming effect comparable to that of a 15 mm-thick down jacket at −20 °C (Figure 6c) [111]. Similarly, heterocyclic aramid nanofibers were used as building units to construct aerogel fibers with a skin-shell–core radial gradient porous architecture (HANF-AFs, Figure 6d). The hierarchical porous architecture suppressed multiple heat-transfer pathways (Figure 6e), while enhancing tensile strength. These combined thermal and mechanical properties enabled HANF-AFs to be woven into thermally insulating textiles, which exhibited excellent thermal retention and cold-protection performance [112]. A more pronounced separation of mechanical and thermal functions was achieved in elastic aerogel fibers encapsulated by a hydrophobic TPU shell (EAF). The nanoporous core provided thermal insulation function, whereas the TPU layer maintained structural integrity during stretching, washing, and repeated weaving. EAFs exhibited a maximum tensile strain of 1600% and were further knitted into an EAF sweater with a thermal conductivity of 26.9 mW·m^−1^·K^−1^. Additionally, the EAF textiles exhibited washability and dyeability that are provided by TPU encapsulation and hold great potential in military uniforms and space suits [101].

When there is a substantial temperature gradient between the surroundings and the skin, radiative heat loss can become an important component of overall heat dissipation. Infrared-reflective components can therefore complement the suppression of conductive and convective heat transfer. Song et al. [113] developed SiC nanofiber aerogels with compressive resilience via chemical vapor deposition. The SiC nanofibers showed high reflectivity and scattering capacity in the MIR band (Figure 6f), enabling the aerogels to reflect infrared radiation emitted by the human body (Figure 6g). Although these aerogels suppress heat loss in cold environments, their wearability remains limited by their mechanical performance. In another study, flexible Kevlar-based aerogel membranes were constructed. Al nanoparticles with high infrared reflectivity (72.77%) contributed to the reflection of infrared radiation emitted by the body, while the 3D porous structures suppressed conductive and convective heat transfer. The aerogel membrane showed a surface temperature approximately 4 °C lower than that of bare skin [114].

#### 4.1.2. Passive Daytime Radiative Cooling

Passive daytime radiative cooling (PDRC) requires simultaneous suppression of solar heat gain and enhancement of radiative heat dissipation to outer space [115]. Therefore, wearable PDRC aerogels should combine high reflectance over the solar spectrum of 0.3–2.5 μm with high emissivity in the atmospheric transparency window (ATW, 8–13 μm) [115,116].

**Figure 7:**
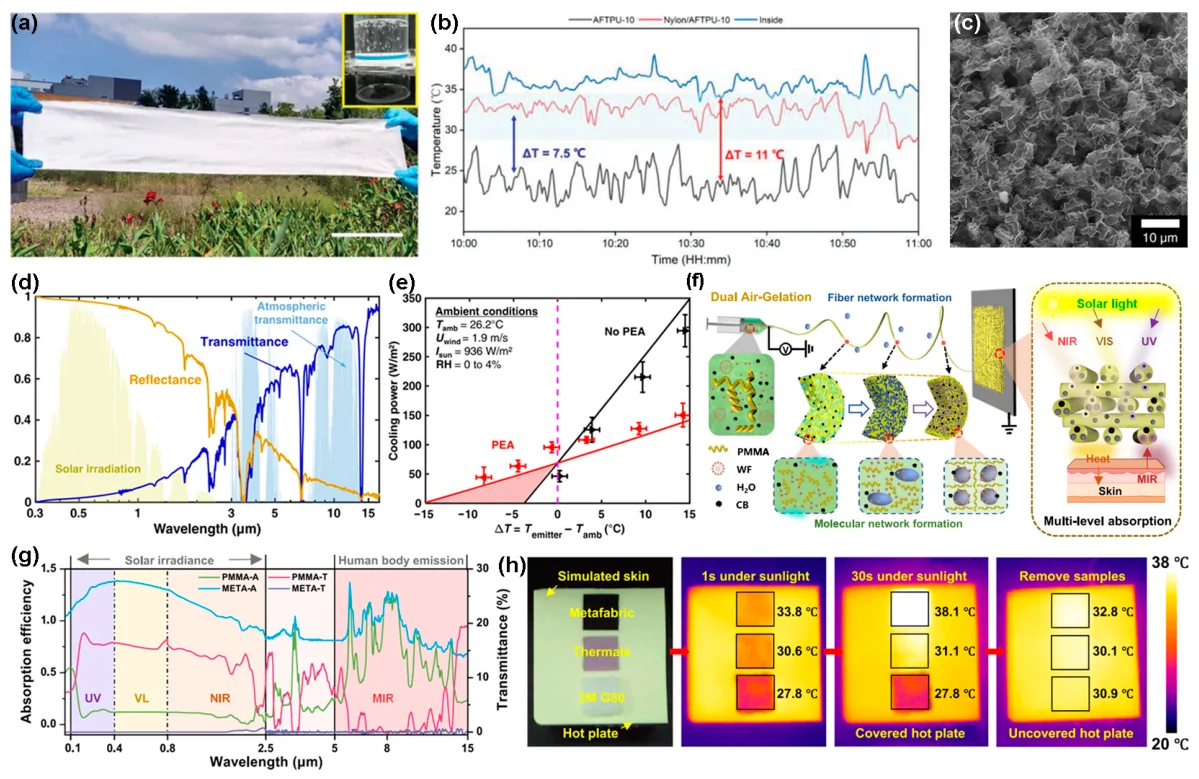
(**a**) Photograph of the front side of nylon/AFTPU-10. (**b**) Time-dependent temperature for skin under different AFTPU-10 and nylon/AFTPU-10 (The blue line showed ambient temperature) in Suzhou, China (October 2021). Reproduced from [117], available under terms of the CC BY 4.0 license, Copyright 2022, Shan X., et al. (**c**) SEM image of PEA. (**d**) Transmittance and reflectance of a 6-mm-thick PEA along with the normalized AM1.5 solar spectrum and the atmospheric transmittance. (**e**) Cooling power of two emitters with 18-mm-thick PEA and without PEA as a function of the emitter subcooling in San Pedro de Atacama, Chile. Reproduced from [107], available under terms of the CC BY-NC 4.0 license, Copyright 2019, Leroy A., et al. (**f**) Fabrication and structure of the fabric for self-sustainable radiative heating. (**g**) Absorptance (A) and transmittance (T) for PMMA aerogel fibrous membranes and metafabrics. (**h**) Optical and infrared images of the hot plate before and after covering different materials. Reproduced from [118], available under terms of the CC BY-NC-ND 4.0 license, Copyright 2024, Tian Y., et al.

Silica-based aerogels provide a representative platform for regulating both spectral regions. The Si-O-Si stretching vibration around 1100 cm^−1^ triggers strong infrared absorption, thereby contributing to high ATW emissivity (8–13 μm). Meanwhile, high reflectance within the solar spectrum can be achieved by modulating the pore structures and skeleton sizes [109]. Li et al. [20] created flexible silica-alumina nanofibrous aerogels (SAFAs) with an ATW emissivity of 83% and a solar reflectance of 95%. Their multilayer porous structures enhanced solar scattering for high reflectance, while the silica–alumina skeleton facilitated high ATW emissivity. The covered body region was maintained at approximately 31 °C, corresponding to a theoretical cooling power of 133.1 W/m^2^. A silica aerogel-functionalized TPU composite film (AFTPU) was developed for high-efficiency PDRC. The composite containing 10 wt% silica aerogels (AFTPU-10) exhibited an enhanced ATW emissivity of 0.96, attributed to the strong infrared absorption of Si–O–Si bonds. AFTPU-10 was further applied as a functional coating on traditional nylon (Figure 7a). When exposed to direct sunlight, AFTPU-10/nylon showed a skin-side temperature of 22.7 °C, lower than the 43.8 °C measured beneath conventional cloth (Figure 7b) [117].

Beyond tailoring spectral properties, hierarchical architectures can spatially distribute solar-reflection, infrared-emission, and mechanical load-bearing functions among different structural components. Hetero-structured fibers and multilayer laminates provide key routes for integrating efficient PDRC with wearability. Jiang et al. [119] designed a tree-ring aerogel fiber comprising a dense cellulose aerogel core and a sheath layer of cellulose aerogels embedded with hollow silica spheres. In the coaxial structure, the hollow spheres further enhanced solar scattering, yielding a solar reflectance of 92.6%, while the cellulose aerogel core offered high tensile strength (19.4 MPa) with sufficient flexibility for textile processing. When covering the arms, the highly breathable fabric achieved a 4 °C temperature reduction compared with cotton fabrics. In a polyethylene aerogel (PEA), high solar reflectance (0.922) and low thermal conductivity (0.028 W·m^−1^·K^−1^) were achieved by a hierarchical porous structure spanning nano- and microscale dimensions (Figure 7c,d). Building upon this optically selective and thermally insulating framework, PEA was further integrated with a black emitter (20 mm thick), yielding a cooling power of 96 W·m^−2^ (Figure 7e). Notably, separating the reflector and emitter into distinct components allows independent optimization of optical and thermal functions [107].

PDRC aerogels can establish stable local thermal environments for health monitoring and therapeutic applications. Zhong et al. [120] proposed a passive isothermal flexible sensor based on cellulose aerogel (HCA), which was formed by entangled hollow cellulose microfibers and bridge-like connections. HCA exhibited a high MIR emissivity of 92.7% and a solar reflectance of 95.6%, attributed to the combined effects of cellulose chemical bonds, multiscale microfiber structures, and a negligible extinction coefficient. An assembled sensor showed a temperature of 29 °C, minimal fluctuations, and a rapid response under direct sunlight. The cooling layer reduced thermal interference during human-motion monitoring and limited heat-induced skin inflammation. Zhang et al. [121] developed a biocompatible polyamide 6/silk fibroin nanofiber dressing that combined a high solar reflectance of 0.96 with a high ATW emissivity of 0.94. The dressing exhibited a temperature drop of 7 °C below ambient temperature. By reducing local heat accumulation, it suppressed oxidative stress and inflammation and consequently promoted wound healing.

#### 4.1.3. Photothermal and Electrothermal Heating

Photothermal and electrothermal aerogels provide external heat input when passive thermal insulation is insufficient to maintain personal thermal comfort. Photothermal heating relies on the absorption of solar radiation and its conversion into heat, whereas electrothermal heating generates Joule heat through a conductive network under an applied voltage [115,122]. The porous aerogel framework can additionally reduce outward heat transfer, accommodate energy-storage components, and maintain mechanical compliance during wearable use.

Solar-thermal conversion can be coupled with latent-heat storage to prolong the heating effect. Sun et al. [123] reported a flexible graphene-based aerogel phase-change film by incorporating paraffin wax into a porous rGO aerogel framework. The strong sunlight absorption capacity of rGO enabled efficient conversion of incident solar radiation into heat, while the paraffin wax absorbed and stored part of the generated thermal energy through phase transitions. The composite film achieved high solar-to-thermal conversion efficiency (95.98%). When attached to the chest of a human-body model under an irradiation intensity of 150 mW/cm^−2^, its temperature increased to 53 °C. The phase change component also delayed temperature decay after removal of the light source, thereby extending the duration of thermal regulation. An ultrathin self-sustainable heating fabric with a 3D double-network structure was prepared through electrospinning and dual aerogelation (Figure 7f). The high optical transparency of polymethyl methacrylate (PMMA) facilitated light penetration through the multilayer structure, while carbon black provided strong broadband absorption of both solar and human-body infrared radiation (Figure 7g). Under simulated sunlight, the fabric produced a stable temperature rise of 8.8 °C on a skin simulator. Within 3 h after the light was removed, it enhanced solar-thermal energy retention by over 71% compared with traditional heating fabrics (Figure 7h) [118].

The integration of conductive nanomaterials endows aerogels with additional heating functionalities, enabling their application in versatile scenarios. He et al. [21] reported a wearable active-cum-passive heater based on kapok fiber/CNTs/calcium alginate aerogels. The rough surface formed by embedding CNTs into hollow fibers facilitated multiple reflections, leading to increased solar absorptance (96.5%). Joule heating was further achieved by the continuous conductive network formed by CNTs. When exposed to a solar lamp (200 W/m^2^) with a room temperature of 11.1 °C, a human body covered by the heater reached a temperature of 45.6 °C. Li et al. [124] developed a biomimetic composite cotton fabric by combining an MXene film with a cellulose aerogel/cotton fabric. The flexible cotton fabric provided a mechanically robust and bendable substrate. The high MIR emissivity and strong electromagnetic wave absorption of the MXene film prevented radiant heat loss from the body and achieved radiant heating, which displays enormous potential in heating clothing and infrared camouflage.

#### 4.1.4. Adaptive and Switchable Thermal Regulation

Adaptive and switchable thermal regulation modulate the local thermal environment under large temperature fluctuations. Such regulation responds to external changes via heating or cooling, ensuring basic thermal comfort for the human body [5]. Based on their operating mechanisms, wearable aerogel systems can achieve this regulation through temporal latent-heat buffering, spatial integration of complementary thermal functions, or switchable spectral modulation.

**Figure 8:**
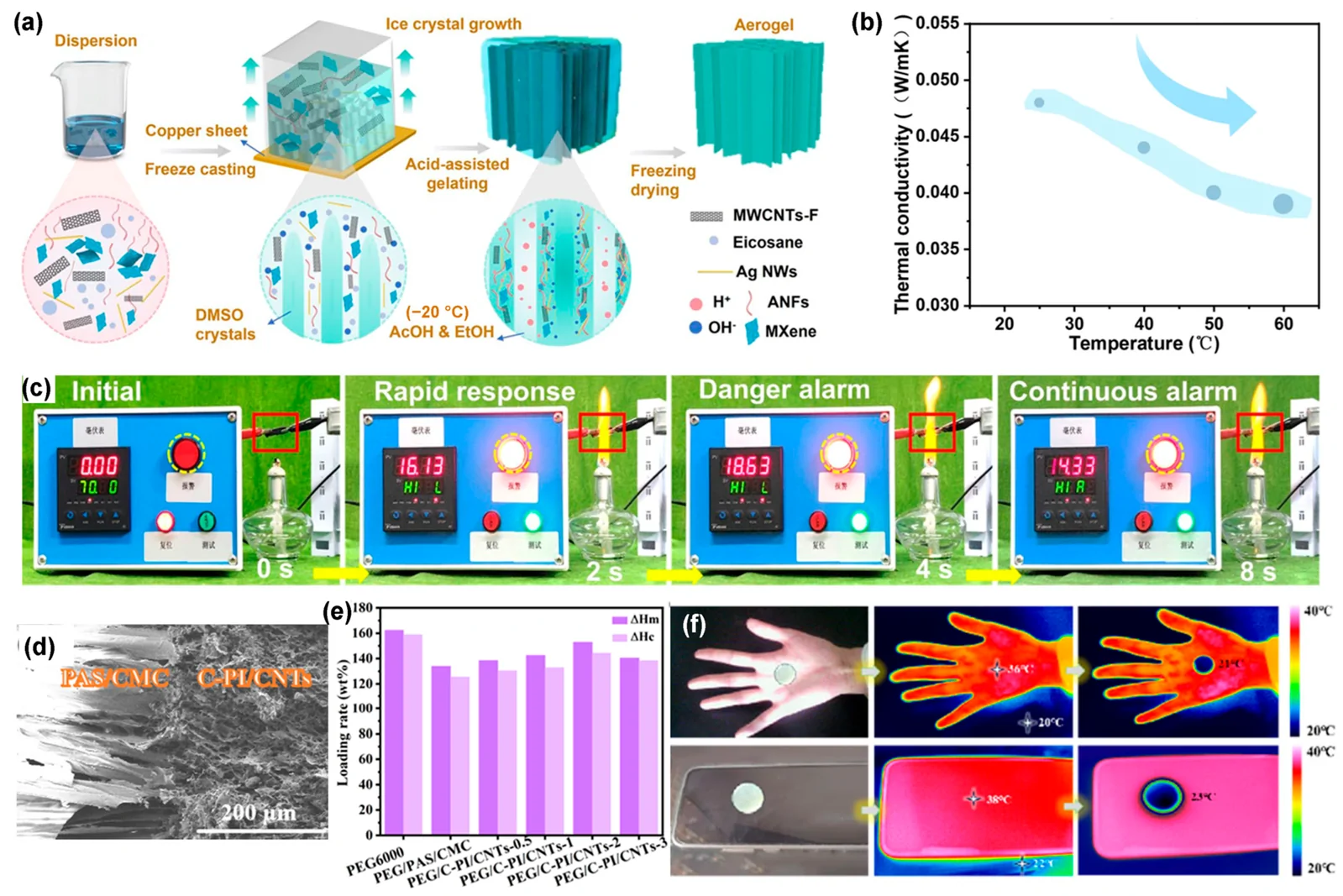
(**a**) Schematic diagram of preparation of the anisotropic ACMCA aerogel. (**b**) Thermal conductivity curve of ACMCA aerogel as a function of temperature. (**c**) High-temperature warning test of ACMCA aerogel when exposed to alcohol lamp flame. Reproduced from [127], available under terms of the CC BY 4.0 license, Copyright 2025, Yu Z., et al. (**d**) SEM image of double-layer aerogels with pronounced interface junctions. (**e**) Enthalpy for PEG and CPCMs. (**f**) Photographs and Thermographic images of poly(ethylene glycol) (PEG)/PAS/CMC@PEG/C-PI/CNTs-2 showing Infrared thermal camouflage when applied to the human body and electronic equipment. Reproduced from [128], available under terms of the CC BY 4.0 license, Copyright 2025, Tian Y., et al.

Phase change materials (PCMs) provide temporal thermal buffering by isothermally absorbing or releasing heat through phase transition. Their integration into aerogels can suppress leakage, maintain structural integrity, and reduce the rate of heat exchange with the surroundings [125]. Phase change aerogels with ordered 3D honeycomb architectures were fabricated via chemical crosslinking and unidirectional freezing. Their hierarchical pores and continuous skeleton that consisted of PDA-coated mineral-based PCM and crosslinked polymers provided high resilience while confining the PCM. During melting, the transition from an ordered crystalline state to an amorphous molten state disrupted the phonon transport path, thereby reducing thermal conductivity and preventing heat loss. These aerogels can be integrated into thermal-storage masks and thermal-regulation wristbands to provide stable local temperature control [126]. Yu et al. [127] developed an ANF/eicosane/conductive component (ACMCA) aerogel by constructing a highly ordered layered porous framework through directional freezing (Figure 8a). In addition to latent-heat buffering, the ACMCA aerogel displayed temperature-responsive thermal-resistance switching, attributed to enhanced interfacial phonon scattering and the resulting decrease in thermal conductivity at elevated temperatures (Figure 8b). These two mechanisms provided complementary thermal protection through latent-heat buffering and temperature-responsive thermal-resistance switching. A conductive MXene/AgNW/CNT network further enabled thermoelectric energy conversion. When integrated into a firefighter uniform, the temperature difference drove directional carrier transport and generated a millivolt-level output voltage, which could trigger the wireless alarm system (Figure 8c).

Spatially heterogeneous structures can assign thermal insulation and heat storage to different regions. Such gradient, bilayer, or Janus architectures allow the dominant thermal-management function to change with environmental temperature, irradiation conditions, or heat-flow direction. Tian et al. [128] developed a bilayer integrated composite (Figure 8d) comprising a thermally conductive carbonized PI/CNT aerogel (C-PI/CNTs) layer and a thermally insulating aerogel layer (PAS/CMC). After PCM impregnation, the composite (CPCMs) combined directional heat transfer, thermal insulation, and latent-heat storage (Figure 8e). When exposed to an external heat source at 55 °C, the insulating layer suppressed heat penetration and maintained the opposite surface at approximately 44 °C. This thermal-shielding capability also reduced the apparent infrared temperature contrast, demonstrating potential for infrared thermal camouflage of the human body and high-temperature equipment (Figure 8f). Su et al. [129] fabricated a Janus-type wearable device consisting of Kevlar nanofiber aerogels impregnated with PCM (KPG) and Kevlar nanofiber aerogels incorporated with hydroxyapatite (HKA). The HKA layer with low thermal conductivity was placed adjacent to the skin, acting as an efficient thermal barrier against heat transfer from the external environment. Meanwhile, the KPG layer absorbed and stored latent heat through phase change, thereby actively buffering temperature fluctuations within the skin microclimate. Through the complementary effects of thermal insulation and latent-heat storage, the device maintained the final skin temperature at 36 °C.

Spectral modulation offers an alternative strategy by regulating solar absorption and thermal emission for adaptive temperature control. Gu et al. [130] reported a hierarchical nanofiber fabric (HNF) integrating radiative-cooling and solar-heating layers. The poly(vinylbutyral) (PVB)-PDMS film featured high solar reflectance (92.2%) and ATW emissivity (90.4%), thereby promoting radiative heat dissipation. In contrast, the polyacrylonitrile (PAN)-CNTs film with a solar absorptance of 80.1% effectively captured sunlight and converted it into heat. By switching the outward-facing layer, the HNF enabled adaptive thermal regulation. The PVB–PDMS side showed a 4.8 °C sub-ambient cooling under hot conditions, whereas the PAN–CNT side generated a temperature increase of 22.9 °C in cold environments. Jiang et al. [131] reported functional cellulose aerogel fibers and fabrics utilizing reversible thermochromic microcapsules. The fabrics adjusted their color by sensing ambient temperatures, further realizing photothermal modulation for temperature control. For instance, the fabric integrated with BKTMs-18 microcapsules (melting point of 18 °C) appeared white at 25 °C. As the temperature declined to its crystallization temperature, the fabric turned black and absorbed 70% of solar radiation. Under illumination, the thermochromic fabric reached a temperature 6 °C higher than that of ordinary fabrics.

### 4.2. Wearable Sensing and Energy-Storage Devices

Flexible aerogels provide deformable porous frameworks for wearable sensing and energy storage. Their structural deformation and accessible interfaces enable mechanical or biochemical signal transduction, while interconnected pores maintain ion and electron transport in electrochemical devices [1]. This section primarily reviews aerogel-based wearable sensors for health monitoring and human–machine interaction, as well as flexible supercapacitors based on aerogel electrodes.

#### 4.2.1. Piezoresistive Strain and Pressure Sensors

Piezoresistive sensors convert mechanical stimuli into resistance variations by changing conductive pathway continuity, conductive contacts, or tunneling distance [132]. When subjected to external loads, highly flexible and resilient aerogels undergo pore deformation, thereby altering their conductive properties and generating electrical signals; upon load release, they return to their original structures, making them promising platforms for highly sensitive sensors. The dominant piezoresistive response depends strongly on the applied loading mode. Tensile strain separates conductive components and induces interfacial sliding or partial disruption of conductive pathways. In contrast, compression collapses pores, generating additional contacts and shortening tunneling distances [133,134].

For tensile strain sensing, the electrical response generally originates from the progressive disruption and reconstruction of conductive pathways during elongation. High sensitivity at small strain can be obtained by concentrating deformation at interfaces or controlled cracks. More importantly, geometrically deformable networks can preserve electrical continuity over larger tensile strains. Zhang et al. [135] reported vanadium nitride (VN)/CNT hybrid aerogels with a sandwich architecture. Vertically aligned CNTs were grown on the VN nanosheets, forming interconnected 3D conductive networks and promoting interlayer charge transfer. Under tension, interlayer sliding and the reversible disruption of conductive contacts produced pronounced resistance changes, achieving ultrahigh sensitivity at strains below 10%. An aerogel-based electronic system (PAPE) was developed by printing a silver (Ag) sensing layer onto a highly stretchable CNF aerogel film. The modulus mismatch between the aerogel substrate and the Ag layer induced numerous microcracks, amplifying resistance variation under tensile deformation. Meanwhile, the rough aerogel surface guided the formation of a distributed and interconnected crack network while suppressing the propagation of through-thickness cracks. This controlled cracking mechanism combined high sensitivity with large strain tolerance. The PAPE electronic devices achieved a gauge factor (GF) of 238 at 10% strain and a low detection limit of 0.1%. When integrated into an e-mask, they can precisely monitor human motion and detect health signals in real time [136].

**Figure 9:**
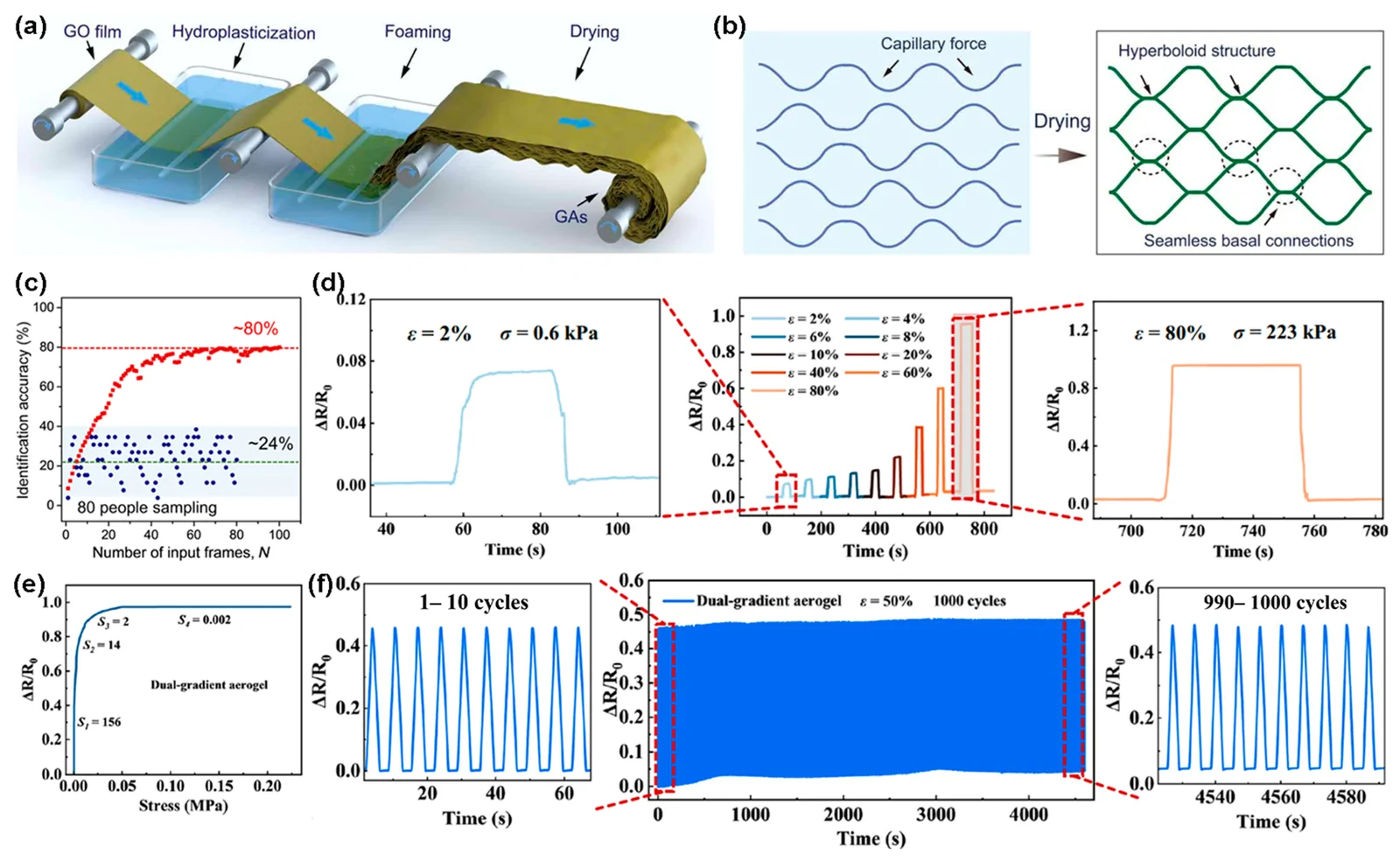
(**a**) Schematic diagram of hydroplastic foaming for the preparation of GO aerogels. (**b**) Seamless basal connections of graphene sheets formed by capillary-driven bubble clustering. (**c**) Classification accuracies of the artificial tactile sensor and human participants based on samples from 80 individuals. Reproduced from [137], available under terms of the CC BY-NC 4.0 license, Copyright 2020, Pang K., et al. (**d**) Time-dependent relative-resistance responses pressure sensor at different compressive strains. (**e**) Relative-resistance change and compressive stress with strains ranging from 2% to 80%. (**f**) The stability of variation in relative resistance of the pressure sensor under compression-unload test for 50% strain and 1000 cycles Reproduced from [134], available under terms of the CC BY-NC-ND 4.0 license, Copyright 2026, Li C., et al.

Under compressive loading, piezoresistive aerogel sensors operate mainly through pore collapse and the progressive formation of conductive contacts. A hydroplastic foaming method was used to convert GO solids into strain-sensing aerogels with a hyperboloid structure (Figure 9a). The seamless basal connections between GO sheets endowed the aerogels with exceptional mechanical stability under large compressive deformation (Figure 9b). Meanwhile, the thin cell walls readily deformed at low strains and generated pronounced resistance variation. This synergistic effect enabled a gauge factor of 1.24 while maintaining an ultra-wide sensing range of 0–95%. A 3D-printed microarray sensor featuring ultrahigh sensitivity and excellent stability achieved 80% accuracy in intelligent touch identification assisted by robotic deep learning (Figure 9c) [137]. Xu et al. [138] constructed CNF/MXene aerogels with a layer-strut cellular structure through an ice-templating method. The MXene-coated fiber layer and the struts mutually supported each other, enabling regular and reversible structural collapse and contact evolution under compression. This controlled deformation maintained a monotonic and stable electrical response. Benefiting from their high electrical conductivity, good compressive recoverability, and cycling stability (1000 cycles), the resulting sensors exhibited high sensitivity and a rapid response. Wrinkled single-layer MXene nanosheets were incorporated into a flexible PI aerogel to build a piezoresistive sensor. This wrinkled morphology provided abundant conductive contact sites and enabled pronounced contact evolution under compression, thereby enhancing the piezoresistive effect. Combined with the high electrical conductivity, this structural design enabled high sensitivity at a high pressure range of 5.3–27.1 kPa and at strains of 60–70% [139].

Although pore deformation and contact evolution enable high piezoresistive sensitivity under compression, maintaining this sensitivity over a broad loading range remains challenging. At low pressures, deformable pores produce rapid changes in conductive contacts. However, complete densification at higher pressures may cause electrical saturation. Therefore, pressure-sensing structures must combine high initial deformability with delayed structural collapse. Gradient structures provide a means of extending the detectable pressure range by regulating the order of pore collapse. PI nanofiber (PINF)@CNT aerogels with gradients in both pore structure and density were prepared by directional freezing of a PINF-based suspension with progressively decreasing layer density. This dual-gradient architecture improved both sensitivity and detection range (Figure 9d). The low-modulus layer underwent pronounced deformation at low pressure, providing high compliance and sensitivity. Under high pressure, the high-modulus layer progressively bore a greater fraction of the applied load, delaying structural and electrical signal saturation and extending the upper detection limit to 223 kPa (Figure 9e). The sensor also maintained a stable response over 1000 compression cycles at 50% strain (Figure 9f) and exhibited reliable sensing performance over a wide temperature range from −196 to 533.3 °C [134]. Similarly, Liu et al. [140] constructed a self-powered flexible pressure-sensitive electronic-skin comprising a low-modulus MXene/PU aerogel anode, a moderate-modulus gel electrolyte, and a high-modulus cathode with microscale protrusions. Under low pressures, the highly deformable anode generated large contact-area variations, ensuring high initial sensitivity. At higher pressures, the high-modulus cathode mitigated resistance saturation and maintained the pressure-dependent response. This modulus-gradient design broadened the pressure detection range. Consequently, the electronic skins (e-skins) could monitor human motions and physiological signals and was also applicable to tire-pressure sensing.

#### 4.2.2. Electrochemical Biosensors

Wearable electrochemical biosensors convert biochemical reactions involving target analytes into measurable electrical signals by integrating biorecognition elements with electrochemical transducers [141,142,143]. In addition to high sensitivity and selectivity, these devices require conformal skin contact, efficient transport of body fluids, and stable operation during bending, stretching, and twisting [1,144]. Aerogels can satisfy these requirements in different ways. They can serve as breathable mechanical substrates for integrated sensor arrays, function directly as porous electrochemical electrodes, or form ion-accessible semiconductor channels in organic electrochemical transistors.

Aerogel-supported e-skins integrate biochemical sensing components onto soft, permeable aerogel substrates. The interconnected 3D capillary channels facilitate heat and the transport of sweat and vapor at the skin–device interface, reducing liquid accumulation and improving wearing comfort. Meanwhile, the recoverable porous skeleton maintains the integrity of the deposited sensing components during repeated deformation. Zhu et al. [145] developed an e-skin by printing sensor arrays onto gelatin methacryloyl aerogel (FGA). The highly interconnected porous structure endowed the FGA substrate with excellent bendability, compressibility, twistability, and shape recovery. For biochemical sensing, the working electrode was functionalized with analyte-specific oxidases. The resulting e-skin enabled simultaneous monitoring of multiple physical, physiological, and chemical signals, including interstitial-fluid glucose and sweat alcohol levels, as well as continuous monitoring of interstitial fluid lactate.

Aerogels can also function directly as electrochemical electrodes by combining large accessible surface areas with continuous ion- and electron-transport pathways. Chen et al. [146] developed a biosensor based on a 3D porous MXene/rGO-based aerogel for monitoring glucose levels and pH fluctuations in sweat. Ti_3_C_2_T_x_ nanosheets distributed within the pores and interlayer spaces of the rGO nanosheets increased the electroactive surface area, improving the electron transfer rate at the electrode-electrolyte interface. The porous hybrid electrode also firmly anchored glucose oxidase (GOx), reducing enzyme leakage and shortening the electron transfer distance between the enzyme redox center and the electrode. These features enabled sensitive, rapid, and selective glucose detection. The sensor maintained its detection capacity after 1000 bending cycles, demonstrating good mechanical durability for wearable applications.

Organic electrochemical transistor (OECT) biosensors are a significant class of electrochemical biosensors. They generally consist of a gate electrode, an electrolyte, source and drain electrodes, and an organic semiconductor film situated between the source and drain electrodes [87,147]. The sensing process can be described as follows: a specific target reacts with the gate or channel, altering the potentials between the gate and the electrolyte, or between the channel and the electrolyte, and generating quantifiable channel current responses [23]. Hu et al. [147] developed highly flexible OECTs based on nanoporous PEDOT:PSS polymer aerogel films. The interconnected pores promoted ion penetration/transport by increasing ion transport pathways and interfacial areas, thereby enhancing the active-layer capacitance and transconductance. A glucose biosensor was fabricated by immobilizing GOx onto the gate electrode. The resulting device achieved a broad detection range of 1 pM–5 mM, while maintaining selective glucose tracing in the presence of interfering species. PEDOT aerogel films fabricated utilizing a uniaxial pre-stretching strategy exhibited ultrahigh tensile strains of 100–200% and strain-insensitive electrical properties. As a dopamine (DA) biosensor, the OECT achieved a low detection limit of 1 nM, with the following sensing mechanism: DA was electro-oxidized on the gate surface, thereby increasing the gate potential and producing a detectable change in drain current. Notably, the biosensor maintained a stable response to DA under 20% strain [87]. This strategy has also been extended to stretchable OECT biosensors for ascorbic acid (AA) detection. The resulting devices enabled precise AA detection in artificial sweat and calf serum, as well as real-time monitoring of salivary AA. Their strong response to AA and weak reactivity to cysteine, glucose, or lactate ensured good sensing selectivity [23].

#### 4.2.3. Flexible Supercapacitors

Supercapacitors are attractive power sources for wearable electronics due to their ultrafast charge–discharge rates and high power density [148]. Their charge storage is generally based on either electrostatic ion accumulation at the electrode–electrolyte interface or rapid surface and near-surface redox reactions [149]. However, two-dimensional electrode materials, such as graphene and MXene, tend to restack during processing, reducing ion-accessible surface area and obstructing electrolyte transport [150]. Aerogel architectures mitigate this limitation by maintaining open 3D networks, exposing electrochemically active interfaces, and providing continuous ion- and electron-transport pathways [27]. Their mechanical continuity also allows the electrodes to retain electrochemical performance during bending, stretching, and textile deformation.

One strategy is to load redox-active phases onto conductive aerogel frameworks. The aerogel framework suppresses the aggregation of active components, maintains continuous electron-transport pathways, and facilitates electrolyte penetration, whereas the incorporated phase increases the density of redox-active sites. A flexible boron-doped graphene aerogel (BGA) and a core–shell CoMoO4@CoP-modified BGA were assembled into a hybrid supercapacitor. The self-supporting BGA electrode exposed more active sites, thereby promoting interfacial redox reactions within the heterojunction. Meanwhile, boron doping altered the electronic structure of graphene and generated multiple additional electroactive regions, enhancing ion/electron transfer rates. Benefiting from these complementary effects, the device achieved an energy density of 50.2 Wh·kg^−1^ at a power density of 800 W·kg^−1^ and retained 95.6% of its capacitance after 10,000 cycles [151]. Guo et al. [152] constructed a 3D porous aerogel electrode by integrating CeCoS_x_ and sodium alginate-doped graphene into a carbonized melamine foam scaffold (CeCoSx−SA/GF). The scaffold suppressed the agglomeration and restacking of graphene nanosheets, thereby preserving the interconnected porous structure and exposing more redox-active sites. This architecture also facilitated electrolyte penetration and ion/electron transport, leading to enhanced electrochemical performance. Consequently, the aerogel cathode achieved a specific capacitance of 873.3 F·g^−1^ at 1 A·g^−1^, while the resulting supercapacitor displayed high power density.

A second strategy is to regulate the spacing and interfacial coupling between two-dimensional nanosheets. A MXene-based aerogel film electrode combined pseudocapacitive and electric double-layer capacitive behaviors. Single-walled carbon nanotubes were intercalated between the MXene nanosheets to suppress self-restacking and establish a highly conductive network. The resulting porous architecture also exposed more active sites and shortened ion diffusion pathways, thereby facilitating reversible proton intercalation/deintercalation and titanium redox reactions within the MXene layer. Consequently, the electrode achieved a high area specific capacitance of 746.68 mF·cm^−2^, together with stable electrochemical performance under bending and prolonged cycling [25]. Heterointerface engineering can further improve ion adsorption and transport while enabling wearable electrode formats. Tang et al. [150] developed wearable fiber-shaped supercapacitors (FSCs) using heterostructured black phosphorus (BP)/MXene aerogels (A−BP/Ti_3_C_2_T_x_) as fiber electrodes. The hierarchically porous structure and strong interfacial interactions between BP and Ti_3_C_2_T_x_ lowered ion-diffusion energy and improved the H^+^ adsorption and transport kinetics, enabling a high specific capacitance (369 F·g^−1^). The FSC achieved an energy density of 6.39 Wh·kg^−1^ and could be integrated into textiles or conformally attached to tissues as an implantable power source. It also retained 88.52% of its initial capacitance under mechanically conformable conditions, displaying good cycling durability.

Representative aerogel-enabled sensing, biosensing, and energy-storage systems are summarized in Table 4. The table compares functional mechanisms, key performance metrics, and the durability of these devices for quantitative comparison.

## 5. Conclusions and Perspectives

As smart wearable devices are increasingly integrated into personal-use scenarios, there is growing demand for flexible materials capable of withstanding large deformations while maintaining structural integrity and functional stability. Against this background, high stretchability has become an increasingly important property alongside fundamental compressibility and bendability. Therefore, this review examines the construction strategies of flexible aerogels under compressive and tensile loading. Across both loading modes, network and interfacial engineering regulates skeleton and junction deformability and stability, while multiscale architectural regulation controls deformation accommodation and load redistribution. This review further discusses the modulation of their mechanical and functional properties and relevant applications in wearable thermal management, sensing, and flexible energy-storage devices.

Based on the aforementioned discussion, we elaborate on the existing challenges of flexible aerogels. (1) Compressibility, elasticity, and cyclic stability of various types of aerogels have been significantly improved through network and structural regulation. Although micrometer-scale pores and polymer incorporation enhance aerogel deformability, they may also introduce trade-offs with other functional properties, such as electrical conductivity and high-temperature thermal-insulation performance. (2) For electrically conductive aerogels, chemical modification can improve deformability only to a limited extent while potentially compromising their intrinsic electrical properties. Geometrically deformable architectures provide additional deformation space, yet balancing stretchability and controllable nanoscale porosity remains challenging. (3) Tensile strength and cyclic stability are still insufficient for applications requiring long-term structural stability and require further improvement. (4) From an industrial perspective, aerogel commercialization is currently concentrated in thermal-insulation products, particularly flexible silica-aerogel blankets [153], while some polymeric aerogel sheets have also become commercially available [154]. In contrast, multifunctional compressible and stretchable aerogel systems for wearable thermal management and electronics still face substantial barriers, such as processing cost and complexity, scalable manufacturing, and long-term mechanical and functional durability. For wearable applications, breathability, skin compatibility, washability, and device integration impose additional requirements for practical deployment.

Based on these challenges, several future directions can be identified. For both compressible or stretchable aerogels, the mechanisms by which chemical and interfacial engineering improves mechanical properties have been comparatively well studied. A systematic understanding of structural formation and structure–mechanics relationships requires further development, particularly at the microstructural level. Consequently, future studies should also integrate interface reinforcement with architectural regulation to balance deformability, strength, and functional retention. For electrically conductive aerogels, greater emphasis must be placed not only on optimizing mechanical properties but also on preserving the continuity of conductive networks during repeated deformation. More broadly, future development should emphasize scalable processing, standardized durability evaluation, and function retention under realistic service conditions in addition to maximizing individual mechanical properties.

## Figures and Tables

**Table 1:** Compressive properties of aerogel and foam lattices fabricated using various 3D printing technologies and ink compositions.

Ink Composition	Microlattice	Mechanical Performance	Ref.
Ascorbic acid/partially reduced GO	Woodpile	Ultrahigh elasticity (at 95% compressive strain) and high compressive strength	[58]
SiC nanowire/silica particles	Square, hexagonal, and triangular lattices	High Young’s modulus for triangular lattice	[71]
GO/CaCl_2_	Woodpile	High elasticity (at 80% compressive strain)	[69]
PAA	Hexagonal honeycomb	Compressibility (70% maximum strain) and high strength (10.2 MPa) at a density of 0.294 g cm^−3^	[70]
GO/R–F/Silica filler	Cubic-like lattice	High resilience (at 50% compressive strain) and high compressibility (90% maximum strain)	[67]
Cellulose	Honeycomb	High compressive modulus of 16.6 MPa at a density of 0.09 g cm^−3^	[72]
GO-based gel ink	Nonplanar and regular structures	Not reported	[74]
CNF/CNC/CaCl_2_	Complex geometries	High mechanical modulus	[75]
CNF	Grid structure	Enhanced elasticity after water absorption	[76]
Kevlar nanofibers	Honeycomb	Compressibility (50% compressive strain) and impact toughness	[77]
MXene	Cube composed of trusses	High elasticity (at 50% compressive strain) at a density of 0.0157 g cm^−3^	[78]
Crosslinked GO (XGO) resin	Octet-truss-gyroid	High elastic modulus	[79]

**Table 2:** Representative tensile properties and deformation strategies of stretchable aerogels.

Aerogel System	Construction Method	Deformation-Enabling Structure	Tensile Performance	Cyclic Durability	Ref.
Mullite nanofiber aerogel	3D reaction electrospinning	Interwoven crimped-nanofiber network	Tensile strain up to 100%	Recovery after 1000 cycles at 40% strain	[17]
ZrO_2_–SiO_2_ nanofibrous aerogel	Two-component off-axial electrospinning	Highly buckled, multi-arched fibrous network	Elongation at break = 150%	Recovery after 500 cycles at 80% strain	[93]
SiC-SiO_x_ nanowire aerogel paper	Ethanol-induced aggregation and drying	Wrinkled laminated nanowire architecture	Tensile strength = 399 kPa	Not reported	[80]
SiC-SiO_x_ nanofiber aerogel	High-temperature vapor deposition	Curly nanowires and interconnected bundles	Elongation at break > 20%	Residual strain = 1% after 100 cycles at 10% strain	[94]
ANF/PVA aerogels	ANF assembly and crosslinking	Highly connected 3D microfibrillar network	Tensile strength = 6.3 MPa	Not reported	[95]
PU-based fibrous sponge	3D electrospinning and thermal crosslinking	Curly fibrous network with semi-IPN	Tensile strength = 1 MPa; Tensile strain > 40%	Negligible residual deformation after 1000 cycles	[98]
rGO/polymer elastomer	Sol–gel processing and hot pressing	Folded and re-entrant cellular structure	Elongation at break = 1250%	No residual elongation after 1000 cycles at 400% strain	[96]
MXene/PVA aerogel	PVA-assisted assembly and hot pressing	Crimped and re-entrant microstructures	Elongation at break = 427%	No plastic deformation after 1000 cycles at 200% strain	[92]
PEDOT:PSS aerogel film	Pre-stretching and freeze drying	Crimped/folded and re-entrant microstructure	Elongation at break = 200%	High recovery ratio = 93–96% after 1000 cycles at 50% strain	[87]
Semiconducting polymer aerogel film	Templating and pre-stretching	Crimped and folded microstructures	Elongation at break > 40%	100% recovery after 1000 cycles at 40% strain	[23]

**Table 3:** Comparison of deformation mechanisms, construction strategies, and wearable relevance of compressible and stretchable aerogels.

Comparison Aspect	Compressible Aerogels	Stretchable Aerogels
Loading mode	Compression	Tension
Dominant deformation modes	Pore closure; skeleton bending, buckling, and rotation	Molecular-chain extension; fiber straightening and reorientation; pore-wall unfolding; node rotation and path opening
Principal structural challenge	Avoiding irreversible skeleton/network damage and permanent densification	Maintaining network continuity against tensile stress concentration and interfacial failure
Network/interfacial strategy	Flexible skeletons or molecular segments and stable yet compliant junctions	Extensible polymer/hybrid networks with robust or dynamic interfaces
Architectural strategy	Mesoscale cellular architectures and programmable lattices for deformation accommodation and load redistribution	Crimped/entangled fibrous networks, pre-deformed cellular structures, and programmable architectures providing geometric redundancy
Key mechanical metrics	Elastic recovery; compressive strength/modulus; cyclic stability	Elongation at break; tensile strength/modulus; cyclic stability
Functional implication under deformation	Preservation of porous structure and thermally and electrically conductive pathways during repeated compression	Preservation of continuous thermal, electrical, and ion/electron-transport pathways during tensile deformation
Representative wearable relevance	Contact-pressure and pressure-sensing scenarios; compression-resilient PTM layers and porous electrodes	Body-motion and strain-sensing scenarios; stretchable PTM textiles, biosensors, and energy-storage electrodes

**Table 4:** Representative aerogel-enabled wearable sensing and energy-storage devices and their key performance metrics.

Device Type	Aerogel System	Functional Mechanism	Key Performance Metrics	Durability	Ref.
Strain sensor	VN/CNT hybrid aerogels	Disconnection and reconnection of CNTs	GF = 135 ± 7 within the 0–5% micro-strain range	Stable response under 1000 stretching cycles	[135]
Strain sensor	CNF aerogel film	Reversible opening and closure of Ag microcracks	GF = 238 under a tensile deformation of 8–10%	No obvious signal attenuation or baseline drift after 2500 cycles at 2% strain	[136]
Strain sensor	Hyperboloid graphene aerogel	Reversible deformation of cell walls and conductive pathways	GF = 1.24; sensing range = 0–95%	Stable sensing response after 10,000 cycles at 90% strain	[137]
Pres-sure/Strain sensor	CNF/MXene aerogels	Reversible contact and separation of MXene-coated CNF layer	GF = 3.13 over 7.5–50% compressive strain	Stable response after 1000 cycles at 33% strain	[138]
Pressure sensor	MXene/PI aerogels	Contact-area variation and tunneling-distance modulation	Sensitivity = 2.65 kPa^−1^; pressure range = 0–27.1 kPa	Stable response after 1000 cycles	[139]
Pressure/Strain sensor	PINF@CNT gradient	Sequential deformation and progressive load bearing of gradient layers	Sensitivity = 156 MPa^−1^ at 0–3.2 kPa; detection range = 0.6–223 kPa	Stable sensing response after 1000 cycles at 50% strain	[134]
Electrochemical biosensor	FGA aerogels	Enzyme-mediated electrochemical sensing on a porous, permeable FGA	Linear glucose detection range = 0–9 mM	Relative resistance change < 20% after 100 bends	[145]
Biosensor	MXene-rGO aerogels	Enhanced electron transfer and enzyme immobilization	Linear glucose detection range = 20–200 μM	Stable detection after 1000 bending cycles at 180°	[146]
Supercapacitor	CoMoO4@CoP-modified BGA	Heterointerface-enhanced pseudocapacitive redox reactions	Specific capacitance = 3056.4 F·g^−1^ at 1 A·g^−1^	95.6% capacitance retention after 10,000 cycles	[151]
Supercapacitor	CeCoS_x_−SA/GF	Porous conductive network with redox-active CeCoSx sites	Specific capacitance = 873.3 F·g^−1^ at 1 A·g^−1^	87.1% capacitance retention after 5000 cycles at 8 A·g^−1^	[152]
Supercapacitor	Hybrid aerogel film	Suppressed MXene restacking and shortened ion-transport pathways	Areal capacitance = 746.68 mF·cm^−2^	Stable under bending/cycling	[25]
Supercapacitor	A−BP/Ti_3_C_2_T_x_	Heterointerface-enhanced H^+^ adsorption/diffusion and electron transport	Specific capacitance = 369 F g^−1^; Energy density = 6.39 Wh·kg^−1^	88.52% capacitance retention after 5000 bending cycles (120°)	[150]

## Data Availability

No new data were created or analyzed in this study. Data sharing is not applicable to this article.
